# Short‐Term Effect of Air Pollution on Out of Hospital Cardiac Arrest (OHCA) in Lombardy—A Case‐Crossover Spatiotemporal Study

**DOI:** 10.1002/gch2.202500241

**Published:** 2025-09-29

**Authors:** Amruta Umakant Mahakalkar, Enrico G. Caiani, Giuseppe Stirparo, Elena Ticozzi, Lorenzo Gianquintieri

**Affiliations:** ^1^ Department of Electronics Information and Bioengineering Politecnico di Milano Milan 20133 Italy; ^2^ Istituto Auxologico Italiano IRCCS S. Luca Hospital Milan 20149 Italy; ^3^ Agenzia Regionale Emergenza Urgenza (AREU) Milan 20126 Italy

**Keywords:** air pollution, distributed lag nonlinear model, DLNM, OHCA, out‐of‐hospital cardiac arrest, risk assessment

## Abstract

Evidence linking short‐term exposure to air pollution, particularly PM_2.5_, PM_10_, NO_2_, O_3_, SO_2_, and CO, with out‐of‐hospital cardiac arrest (OHCA) risk remains inconsistent. Large‐scale studies using advanced air pollution exposure assessment techniques and spatiotemporal modeling are limited. This study investigates the association between short‐term air pollution concentration, derived from satellite data, and OHCA events (2016 ‐ 2019) in Lombardy, Italy, using a time‐stratified case‐crossover design. A two‐stage distributed lag non‐linear model, adjusted for meteorological factors, long‐term trends, seasonality, intraweek patterns and holidays, is applied across 96 districts to estimate the exposure‐lag‐response relationship, followed by a regional meta‐analysis. Subgroup analyses by age and sex of OHCA patients, with models stratified by urban‐rural areas and seasons, and interaction with co‐pollutants, are conducted. Among 37,613 OHCA cases, significant associations between OHCA and air pollutants are observed: NO_2_ shows a delayed effect at lag 4 per 10 µg m^−^^3^ increase (RR: 1.071; 95%CI: 1.029–1.114), while PM_2.5_ (RR: 1.029; 95%CI: 1.006–1.053), PM_10_ (RR: 1.025; 95%CI: 1.005–1.046), and O_3_ (RR: 1.009; 95%CI: 1.001–1.016) have immediate effects at lag 0 or lag 1. CO exhibits a modest association per 100 µg m^−^^3^ increase (RR: 1.007; 95%CI: 1.000–1.013, lag 4). Risks are elevated in summer months, and varied by urban‐rural setting. No significant effect modification is detected across age or sex subgroups. Bi‐pollutant models reveal notable interaction and confounding patterns.

## Graphical abstract

This study assesses short‐term air pollution effects on out‐of‐hospital cardiac arrest in Lombardy (2016–2019) using satellite‐derived exposures and case‐crossover design (distributed lag nonlinear model). NO_2_, PM_2_._5_, PM₁₀, O_3_, and CO show significant associations, with varying lags. Risks are stronger in summer and differed by urban–rural context, but not by age or sex.

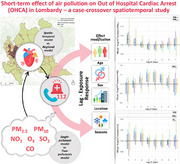

## 1 Introduction

The World Health Organization (WHO) identifies air pollution as the second leading risk‐factor for non‐communicable diseases.^[^ ^1^ ^]^ Its guidelines offer quantitative, health‐based recommendations by providing suggested daily limits for key air pollutants, beyond which there is a significant risk to human health. In recent decades, there has been a global effort to implement various policies aimed at improving air quality. However, the WHO reports that most of the global population remains exposed to air pollutants that exceed its recommended guidelines. Epidemiological studies have established that short‐term exposure to air pollution contributes to increased morbidity and mortality risk from non‐communicable diseases, largely through its effects on cardiovascular and pulmonary systems.^[^ ^2^, ^3^ ^]^

To accurately account for spatial variations in exposure‐response relationships, environmental epidemiological research is increasingly applying advanced exposure measurement techniques for air quality models such as earth observation, chemical transport modeling, machine learning,^[^ ^4^, ^5^ ^]^ and high‐resolution exposure modeling,^[^ ^6^ ^]^ and using advanced spatiotemporal statistical models. In particular, the distributed lag model (DLM)^[^ ^7^ ^]^ has progressively become the state‐of‐art in the field.^[^ ^8^ ^]^

Evidence on association between short‐term air pollution and specific pathologies, such as Out of Hospital Cardiac Arrest (OHCA), remains limited and inconclusive.^[^ ^9^, ^10^ ^]^ OHCA is clinically defined as the loss of functional cardiac biomechanical activity in association with an absence of systemic circulation, occurring outside of a hospital setting. Given the time sensitive nature of OHCA, the preparedness and prompt response of the local emergency medical services (EMS) are crucial in minimizing the risk of asystole caused by cardiac exhaustion and improving overall survival rates,^[^ ^11^ ^]^ making it a particularly sensitive topic. As reported by the European Resuscitation Council, the annual incidence of OHCA in Europe ranges from 67 to 170 cases per 100 000 inhabitants, with a hospital discharge survival rate of 8%, highlighting a pressing public health issue.^[^ ^12^ ^]^ Several risk factors have been linked to cardiovascular diseases that may trigger cardiac arrest, with emerging evidence connecting to short‐term exposure to air pollution.^[^ ^9^, ^10^ ^]^ The existing literature on the relationship between acute effects of air pollution and OHCA reports a positive link between elevated PM_2.5_ levels and increased OHCA risk,^[^ ^13^, ^14^, ^15^, ^16^, ^17^, ^18^, ^19^, ^20^, ^21^ ^]^ while findings for O_3_, SO_2_, NO_2_, and CO are inconsistent.^[^ ^9^, ^10^, ^16^, ^18^, ^19^, ^22^, ^23^ ^]^ Furthermore, with a few exceptions,^[^ ^19^, ^24^ ^]^ most studies feature small sample sizes, gross modeling, or shorter study periods. For instance, some studies^[^ ^24^, ^25^ ^]^ demonstrated a significant positive association using a spatiotemporal distributed lag non‐linear model (DLNM); however, their analyses relied on a rudimentary exposure measurement technique. In contrast, Tobaldini et al.^[^ ^17^ ^]^ applied a DLNM with an advanced chemical transport grid model for pollution estimation, but focused exclusively on particulate matter (<10 µm) with a relatively small sample size of 5761 cases on a homogeneous metropolitan area.

Accordingly, this study aims to examine the exposure‐lag‐response relationship between the daily mean concentration of PM_2.5_, PM_10_, O_3_, NO_2_, CO, and SO_2_ and the incidence of OHCA recorded by the prehospital EMS in the Lombardy region from January 2016 to December 2019, using a spatiotemporal statistical model. Surges in air pollution have been linked to increased ambulance dispatches for cardiovascular diseases, including cardiac arrests,^[^ ^9^ ^]^ making prehospital emergency data particularly valuable for investigating this association. Furthermore, the study compares the results of the spatiotemporal model with a simplified regional aggregated model, to understand the influence of spatial heterogeneity on risk. While pollutant levels are typically higher in larger cities, the chemical composition of particulate matter, along with population characteristics and access to healthcare services, can differ substantially from rural areas. These variations in vulnerability across populations and territories are crucial to understand.^[^ ^3^ ^]^ Therefore, this study also investigates the influence of potential effect modifiers such as age, sex, seasons, and location on OHCA risk through stratified modeling, along with multi‐pollutants interactions, which could be potentially confounders.^[^ ^2^, ^8^ ^]^

## 2 Methods and Data

### 2.1 Site Context

Lombardy, located in northern Italy, is the country's most populous region and one of Europe's densest territories. With a population of 10 million across 23844 km^2^, Lombardy is bordered by the Alpine Mountains ranges to the north, while the cities lie in the plains. The region experiences primarily humid subtropical climate in the south but also have humid continental and temperate oceanic in the north, in the Alpine foothills.^[^ ^26^ ^]^ Due to the varied climatic features driven by geography and land uses, the region also faces hot summers and cold winters with Milan and surrounding cities experiencing an increasing risk of heatwaves.^[^ ^27^ ^]^ The low plains of the Po River valley, enclosed by mountains on three sides, are characterized by conditions that hinder the dispersion of ambient air pollution.

With a high rate of urbanization, primarily driven by the Milan metropolitan area, along with extensive areas under intensive agriculture and industrial activities, Lombardy suffers from episodic surges in air pollution every year.^[^ ^28^, ^29^ ^]^ Although the pollution levels are not increasing rapidly, the region remains one of the most polluted in Europe, with significant consequences for public health.^[^ ^30^ ^]^ A thorough description of pollution spatial‐temporal patterns (including seasonality) in the Lombardy region is provided in a previous study based on the same air pollution dataset.^[^ ^29^ ^]^

### 2.2 Exposure Data

The environmental data for PM_2.5_, PM_10_, NO_2_, O_2_, SO_2_, and CO from January 2016 to December 2019 were extracted from the Copernicus Atmosphere Monitoring Service (CAMS) reanalysis database.^[^ ^31^ ^]^ The raster data provided surface‐level hourly concentrations of the air pollutants at a spatial resolution of 0.1° x 0.1° (approximately 10 km x 10 km) in µg m^−3^ unit. The ensemble median model is calculated from the individual outputs of nine air quality data assimilation systems across Europe and is validated against the observations from ground stations. The CAMS dataset contained no missing values, as data were continuously recorded and thoroughly pre‐processed.

**Figure 1:**
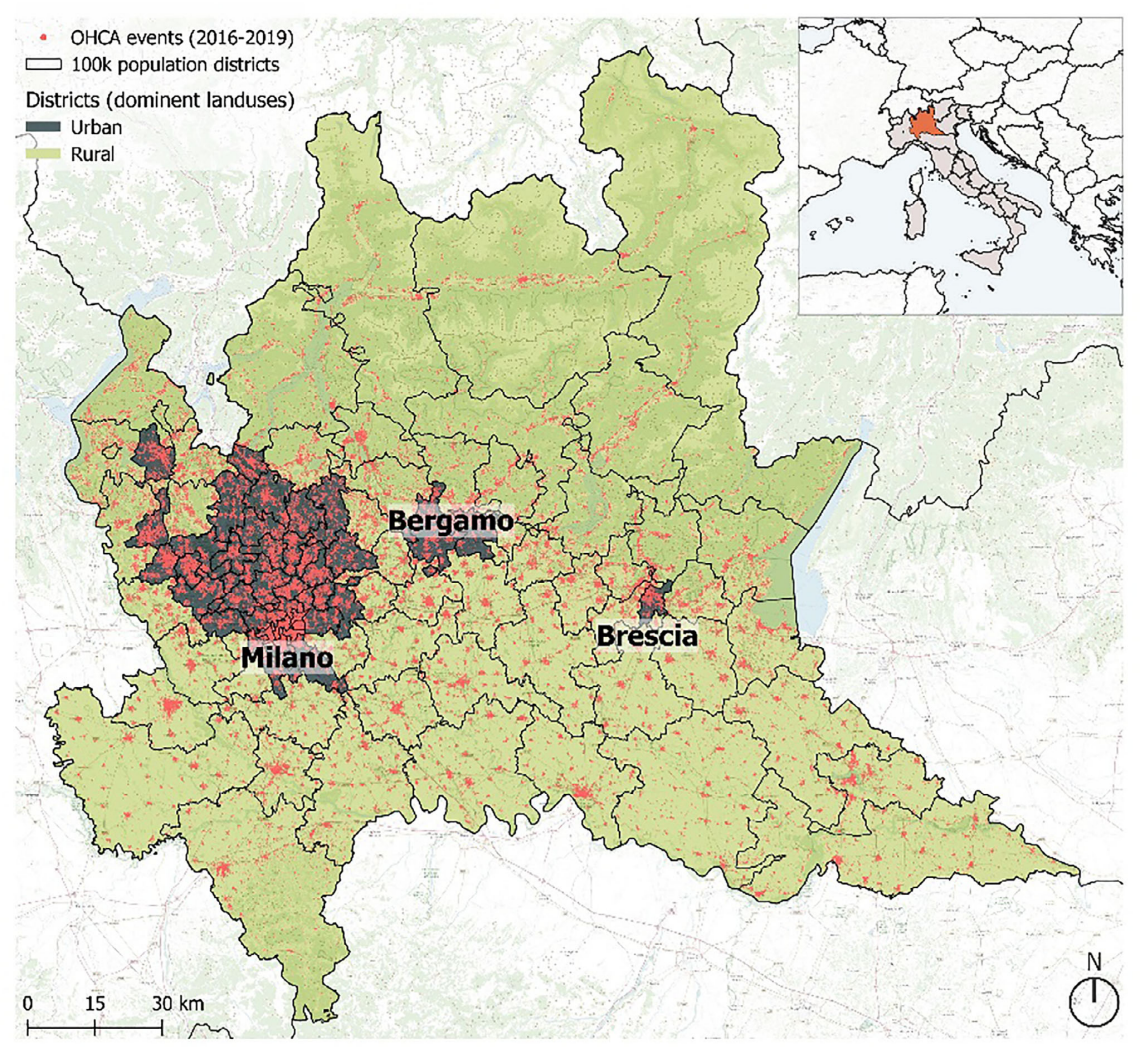
OHCA events in Lombardy region, Northern Italy, from 2016 to 2019, with the demarcation of the three main cities in the region and the boundaries of the 96 districts with approximately 100 000 population utilized in the analysis, and their urban (in red)/rural (in green) characterization. The districts were classified based on their urban land cover with urban areas exceeding the regional mean.

Similarly, time‐varying meteorological confounders (temperature and relative humidity) for the same period were collected from the Copernicus European Regional Reanalysis datasets^[^ ^32^ ^]^ that provide measurements 2 metres above the surface with a temporal resolution of three hours and a spatial resolution of approximately 11 km x 11 km. All pollution and meteorological inputs were aggregated into daily values and spatially averaged for the entire region, as well as within sub‐regional units (districts) with approximately 100 000 residents. The spatial delineation was designed to ensure uniform population density while introducing geographical granularity in the region that is exposed to a wide range of air pollution and meteorological conditions due to its distinct climate zones and dominant land uses. These spatial units were delineated by progressively clustering neighboring municipalities using the methodology described (and validated for environmental epidemiology analyses) in our previous research,^[^ ^33^ ^]^ thus resulting in 96 territorial units (**Figure** **1**).

### 2.3 Health Outcome Data

Geo‐localized data on OHCA events were provided by the Lombardy regional prehospital eEMS provider, Agenzia Regionale Emergenza/Urgenza (AREU). As the data source relies on telephone calls for cardiovascular events, which surged during the first wave of COVID‐19 from early 2020, alongside respiratory emergencies, the study period was limited between January 2016 and December 2019. The data cleaning and filtering methodology is described in S.1 (Supporting Information).

Demographic details of the individuals such as their age and sex, type of call location (home, work, school, public place, etc.), the time between the call and ambulance arrival at the location, the time between ambulance pickup and arrival to the hospital (if admitted), were available. The points corresponding to OHCA events were aggregated in space at the district level and for the entire region, aggregated temporally as daily estimates, and joined with the respective pollution concentrations and meteorological factors in the occurrence area. As the OHCA events are fully anonymized and spatiotemporally aggregated, no specific ethical approval was deemed necessary.

### 2.4 Statistical Model

This study accounts for the spatial heterogeneity of the variables by adopting a two‐stage model, where the exposure‐response models for the individual territorial units are pooled through meta‐analysis to generate risk estimates for the entire region.

In the first stage, a quasi‐Poisson generalized non‐linear regression was fit using a distributed lag non‐linear model (DLNM) as proposed by Sera and Gasparrini^[^ ^34^ ^]^ while adjusting for temperature and relative humidity and controlling for the long‐term trends, seasonality and national holidays. The time‐stratified case‐crossover design was applied to each of the 96 territorial units by referring to the exposure levels of the same case before the event, based on temporal factors: year, month, and day of the week (DOW) that would allow the case to serve as their own control, and account for long‐term trends, seasonality of undefined time‐varying confounders and intra‐week patterns.^[^ ^35^, ^36^ ^]^

(1) $$Y_{t}∼\mathrm{Quasi}-\mathrm{Poisson}\left(\mu_{t}\right)$$

(2) $$\begin{array}{ccc} \mathrm{Log}\left(\mu_{t}\right) & = & \alpha +cb\left(\mathrm{pollutan}t_{t},s_{1},l_{1},df_{1}\right)+cb\left(\mathrm{tem}p_{t},s_{2},l_{2},df_{2}\right),\\ & & ob\left(rh_{t},s_{3},df_{3}\right)+\mathrm{holida}y_{t} \end{array}$$

where *Y_t_* represents the number of cardiac arrests on day *t, µ_t_* is the expected number of cardiac arrest cases due to the environmental exposures at day *t*, *α is* the intercept, *cb (*pollutant_t_ *, s_1_, l_1_)* is the crossbasis of pollutant with transformation curve, *s*, lag, *l* and degree of freedom, df. This bi‐dimensional crossbasis structure is implemented in the form of a matrix, with one dimension representing the exposure and the other representing lag. Temperature (*temp_t_*) and relative humidity (*rh_t_*) are also transformed with appropriate lags using crossbasis and onebasis functions, respectively, with parameters based on sensitivity analyses.

The effect estimates, consisting of coefficients and covariances of exposure‐response and lag‐wise models, were recorded for each district and then used in the second stage of a pooled random‐effect meta‐analysis for the overall region. As a result, individual relative risks (RR) per 10 µg m^−3^ increase in PM_2.5_, PM_10_, NO_2_, and O_3_ were derived from lag 0 to lag 7. Given that daily average SO_2_ concentrations remained below 15 µg m^−3^ throughout the study period, whereas CO concentrations varied in the range of hundreds, the lag‐wise RR was scaled to reflect realistic exposure increment with RR per 1 µg m^−3^ increase in SO_2_ and 100 µg m^−3^ increase in CO. Additionally, exposure‐response curves were generated for each pollutant based on the results of sensitivity analyses, expressed as RR from zero to the maximum recorded value. Results were considered statistically significant at *p‐value* < 0.05 with confidence intervals (CI) of 95% not spanning the null value of 1.

Furthermore, this base model was stratified by the urban or rural characteristics, classifying districts with above or below average urban land cover^[^ ^37^ ^]^ as urban or rural, respectively. Effect modification by age (<65 vs ≥ 65 years), and sex (male vs female) was assessed across the region. Model stratification by urban‐rural division and season was performed to compare pollutant effects during Winter (Dec‐Feb), Spring (Mar‐May), Summer (Jun‐Aug), and Autumn (Sep‐Nov). Exposure–response curves and lag effects relationships were studied within each subgroups with ≥ 65 years (age), male (sex), urban (location) and winter (season) as reference categories. The statistical difference in effect estimates across strata were evaluated using 2‐sample z‐test.^[^ ^38^ ^]^

Based on the results from correlation between pollutants and their time‐series analyses, primary single pollutants models were extended into bi‐pollutant models, with a second pollutant included in the model, to understand the influence of the co‐pollutant concentration on the effect estimates of the primary pollutant.^[^ ^39^ ^]^ The co‐pollutant was either included as a confounder or added as an interaction term, allowing the evaluation of both potential confounding and effect modification on OHCA risk. The lag‐wise heterogeneity of effect estimates between the two was assessed through nested models: single and bi‐pollutant (interaction and confounder), with null hypothesis that there is no difference between the two models (*p* for heterogeneity < 0.05).

Lastly, to analyze the impact of including spatial variability in the model, the results for the overall population and sub‐groups of the two‐stage spatiotemporal model were compared to a simpler DLNM model^[^ ^34^ ^]^ developed using aggregated health outcomes and averaged values assuming a uniform exposure for the entire region of Lombardy.

The data were pre‐processed, and results were mapped using Python 3.11; the models were developed in R (version 4.3.0, primarily using dlnm, spline, gnm, and mixmeta libraries).

#### 2.4.1 Sensitivity Analysis

To ensure the robustness of the findings, different model assumptions including degrees of freedom of smooth functions of time and exposure variables, and lag structures of the exposure variable were assessed using a grid search. This approach allowed dynamic selection of parameters for each variable (S.4, Supporting Information). The parameters were evaluated based on changes in estimated relative risks and overall performance using a modified Akaike's information criterion for quasi‐Poisson (Q‐AIC). Preference was given to parameter combinations that yielded the lowest value of Q‐AIC along with stable and interpretable effect estimates.^[^ ^40^, ^41^ ^]^

First, meteorological confounders, temperature and relative humidity, were examined using a range of smoothing and lag structures informed by existing literature.^[^ ^41^, ^42^, ^43^, ^44^ ^]^ For temperature, knots in its crossbasis were placed towards the tails of the distribution, using combinations of 10^th^, 75^th^ and 90^th^ percentiles. A 14 days lag structure of temperature was evaluated with two functions: stepwise function (strata) and logknots with two knots at equally spaced values in the logarithmic scale. Relative humidity was modelled using a natural cubic spline applied to same‐day values and three‐day running means, with 3 and 4 degrees of freedom, to explore varying levels of smoothness.

Transformation parameters of pollutants were studied by lag length, variable and lag functions. The predictors were assessed with linear and natural spline transformation functions, with degrees of freedom ranging between 2 and 4 for natural spline function. Effect estimates by individual and cumulative lags of 3, 7, 10, and 14 days were tested while comparing two functions: natural spline with 3 degrees of freedom and logknots with two knots at equally‐spaced quantiles.

Furthermore, given the high correlation between PM_2.5_ and PM_10_, the bi‐pollutant models focused on PM_2.5_, NO_2_, O_3_, SO_2_, and CO. The co‐pollutants were evaluated in two ways, as a confounder by adjusting the main model for the co‐pollutant, and as an effect modifier by constructing an interaction term, both using a crossbasis structure of the co‐pollutant.^[^ ^39^, ^41^, ^45^ ^]^ Lastly, models with combinations of long‐term temporal splines, individual day‐of‐week, and holidays in addition to the time‐stratified stratum were compared.

## 3 Results

### 3.1 Exploratory Data Analysis

A total of 37613 OHCA cases were reported in Lombardy between 2016 and 2019 (**Table** **1**), with an average of 25.74 daily cases. Out of the total, 21578 patients (57.37%) were male and 16035 (42.63%) were female. The median age was 79 years, with 7852 (20.88%) cases under 65 and 29761 (79.12%) cases over 65 years of age. Urban areas, covering only 4.25% of the region, produced 53.11% of cases, whereas rural areas contributed for 46.89%. The highest number of OHCA incidents occurred in Winter (11258), followed by Autumn (9232), Spring (9072), and Summer (8051).

**Table 1:** Description of the environmental exposures and study population.

Variable	Total	Median	p25	p75	IQR	Min	Max	WHO limit [µg m^−3^]	Days surpassing WHO limits
PM_2.5_		18.06	12.37	27.07	14.71	3.13	73.59	15.00	63.31
NO_2_		23.11	15.99	34.28	18.29	7.48	66.05	25.00	44.56
PM_10_		22.73	15.68	32.66	16.97	4.01	82.09	45.00	11.02
O_3_		48.18	19.60	73.58	53.98	3.49	130.50	100.00	5.75
SO_2_		2.17	1.70	2.68	0.98	0.58	4.79	40.00	0.00
CO		264.36	213.55	366.96	153.42	120.53	817.27	4000.00	0.00
Temperature		10.77	4.39	17.69	13.30	−6.77	27.06		
Relative humidity		74.36	64.14	83.30	19.16	25.15	98.08		
Total OHCA	37613	25	21	30	9	0	59		
Adult	7852	5	4	7	3	0	14		
Senior	29761	20	16	24	8	0	51		
Male	21578	15	12	17	5	0	33		
Female	16035	11	8	13	5	0	29		
Urban	19977	13	11	16	5	0	35		
Rural	17636	12	9	14	5	0	29		

During the study period, as shown in Table 1, daily concentrations of PM_2.5_, PM_10_, and NO_2_ surpassed their corresponding WHO daily permissible limits in 63.31%, 11.02%, and 44.56% of the considered days, respectively. SO_2_ and CO remained well below WHO limit throughout the study period. Time series analyses from a previous work,^[^ ^29^ ^]^ showed NO_2_ and SO_2_ underwent a steady decline since 2016. PM_2.5_, PM_10_, NO_2_, SO_2_, and CO peaked in Winter and Autumn, while O_3_ peaked in Summer and Spring (May‐August).

**Figure 2:**
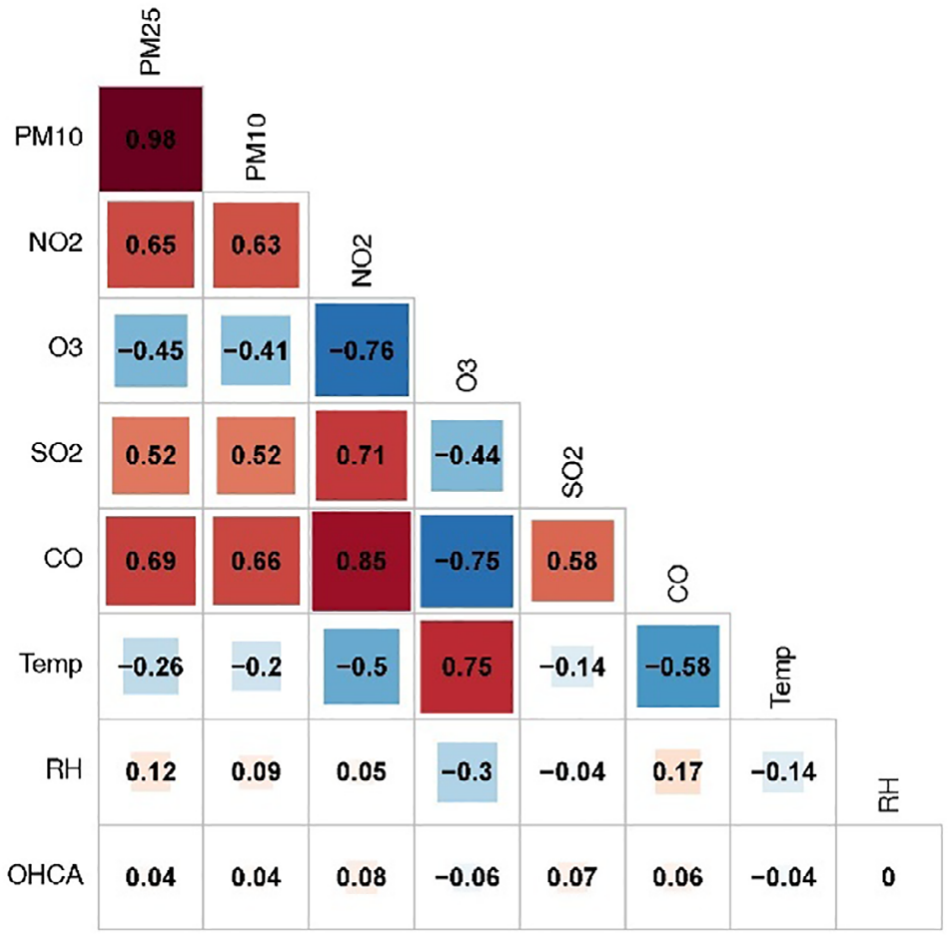
Correlation among environmental exposures and with OHCA in Lombardy region, Italy, from 2016 to 2019.

In terms of the overall Spearman correlation of the pollutants with one another, PM_2.5_ and PM_10_ were found to be highly correlated (*ρ* = 0.98), followed by NO_2_ and CO (*ρ* = 0.85). Ozone, on the other hand, was inversely correlated with all the other pollutants having the highest inverse correlation with NO_2_ (*ρ* = ‐0.76) and CO (*ρ* = ‐0.75). Amongst the confounding variables, temperature was found positively correlated with O_3_ (*ρ* = 0.75) and inversely correlated with CO (*ρ* = ‐0.58) and NO_2_ (*ρ* = ‐0.50), whereas relative humidity was found weakly correlated to all the variables (**Figure** **2**).

### 3.2 Exposure–Lag–Response Relationship

**Figure 3:**
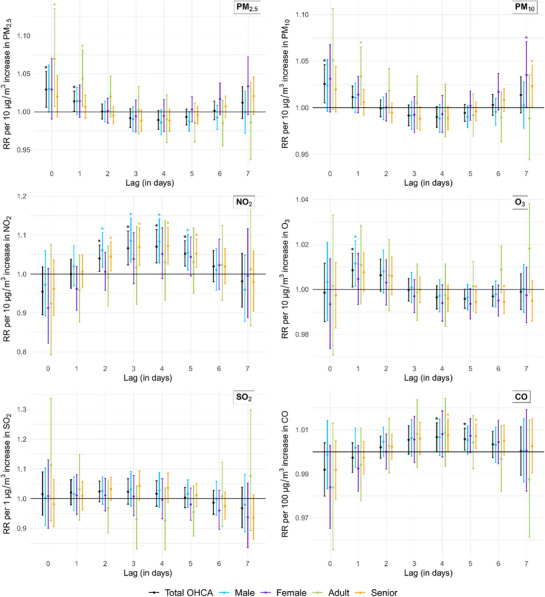
Effect modification by age and sex as relative risk (RR) per 10 µg m^−3^ increase in PM_2.__5_, PM_10_, NO_2_, O_3_; per 100 µg m^−3^ increase in CO; and per 1 µg m^−3^ increase in SO_2_, with 95% confidence interval. *Statistically significant relative risk at *p*‐value < 0.05. No statistical effect modification was observed across subgroups.

From lag‐response diagrams (**Figure** **3**), it was observed that the main spatiotemporal models, adjusted for temperature and relative humidity, showed a positive significant association between OHCA risk and exposures to PM_2.5_, PM_10_, O_3_, NO_2_, and CO for the entire population. The exposure‐response curves demonstrated that the relationship of OHCA risk with pollutants was linear for O_3_ and CO, and non‐linear for the rest (Figures S2– S4, Supporting Information).

The highest risk association was found for NO_2_ at lag 4 with RR: 1.071; 95%CI: 1.028–1.114 per 10 µg m^−^^3^ increase in the NO_2_ exposure, and the relationship stayed significant between lag 2 to lag 5. Meaningful associations were also found for PM_2.5_ and PM_10_ both displayed immediate risk at lag 0 with PM_2.5_ (RR: 1.029; 95%CI: 1.006–1.053), and PM_10_ (RR: 1.025; 95%CI: 1.005–1.046) per 10 µg m^−^^3^ increase, whereas CO had a lower but significant relative risk at lag 5 (RR: 1.006; 95%CI: 1.001–1.011) per 100 µg m^−^^3^ increase. Ozone had significant association with the OHCA risk in the earlier lags of day 1 and day 2 with strongest effect per 10 µg m^−^^3^ increase at lag 1 (RR: 1.009; 95%CI: 1.001–1.016). SO_2_, on the other hand, did not show any risk per 1 µg m^−^^3^ increase, whereas a noticeably high risk per 10 µg m^−^^3^ emeregd at lag 4 (RR: 1.24; 95%CI: 1.029–1.490). Given the improbability of rising SO_2_ by 10 µg m^−^^3^ in the region, the subsequent lag‐exposure assessments were made at the scale of RR per 1 µg m^−^^3^ increase in SO_2_.

The effect estimates demonstrated peculiar patterns when the model was stratified based on urban and rural areas. SO_2_ was found to be significantly associated only in the rural districts (RR: 1.1311; 95%CI: 1.0035–1.2749, lag 0) per 1 µg m^−^^3^ increase. The relative risks of rest of the pollutants remained significant and increased further in the urban areas as compared to the main model. CO, like in the main model, continued to show no risk by urban‐rural stratification. However, the effect modification between urban and rural districts was not significant as per the 2‐sample z‐test (Table S2, Supporting Information).

In contrast, stratifying by seasons demonstrated significant effect modification during summer in reference to winter. The significant risk per 10 µg m^−^^3^ increase was associated with PM_2.5_ at lag 1 and lag 2 (RR: 1.092; 95%CI: 1.008–1.183). Likewise, increase in risks was observed for O_3_ during spring month and CO during winter, but were not significantly different (Table S3, Supporting Information).

The regional model, which assumes a uniform daily exposure across the entire region, produced results that were distinct yet comparable to those of the spatiotemporal model (Figure S5, Supporting Information). In alignment with the spatiotemporal model, the regional model also exhibited significant associations for all the pollutants, with similar lag patterns. Unlike the spatiotemporal model, SO_2_ was significant from lag 3 to lag 5 per 1 µg m^−^^3^ increase, with largest relative risk at lag 4 (RR: 1.0646; 95%CI: 1.0104–1.1217), albeit not significantly different from the effect observed in the spatiotemporal model.

The rest of the pollutants also showed significant positive associations with PM_2.5,_ PM_10_, and O_3_ having strongest associations for lag 0 and lag 1 with comparable effect sizes of (RR: 1.0287; 95%CI: 1.0041–1.0539), (RR: 1.0220; 95%CI: 1.0000–1.0444) and (RR: 1.0104; 95%CI: 1.0027–1.0182) per 10 µg m^−^^3^ increase in their exposure, respectively. On stratification by urban‐rural division, the effect of PM_10_ and O_3_ for urban districts disappeared in the aggregated model, while the effect due to CO remained significant only in the rural districts.

### 3.3 Effect Modification by Age and Sex

After accounting for age and sex in the spatiotemporal model, higher risks of OHCA due to PM_10_, NO_2_, and CO were observed for individuals over the age of 65 years, with significant effect at lag 7 for PM_10_, unlike in the case of the model considering total population. However, the effect modification was not significant in reference to younger individuals. Among those under 65 years of age, significant association to OHCA was observed only for PM_2.5_ and PM_10_ and was marginally higher than that for the total population. Likewise, among females, only PM_10_ showed a larger relative risk, but the effect emerged at a delayed lag of day 7 (RR: 1.0351; 95%CI: 1.0007–1.0707). However, this was not significantly different with reference to males. NO_2_ and O_3_ were more consistently linked to OHCA in males, with a higher effect size than in the overall population. SO_2_ showed no meaningful association in any demographic sub‐group (Figure 3).

To compare the results with the regional model, significant associations to OHCA were found for O_3_ and SO_2_ in addition to NO_2_ for senior population. However, the delayed effect of PM_10_ was not observed among the senior group through this model. O_3_ had an effect among adults, apart from PM_2.5_ and PM_10_. Significant effect estimates among males and females were observed for air pollutants such as PM_2.5_, NO_2_, and SO_2_, which was not observed in the spatiotemporal model. Statistical comparisons across sub‐groups within models, as well as between models applied to different sub‐groups, revealed no evidence of effect modification. Detailed descriptions and remarks are reported in Figure S5 (Supporting Information).

### 3.4 Bi‐Pollutant Models

In bi‐pollutant models, OHCA risk was evaluated for PM_2.5_, NO_2_, O_3_, SO_2_, and CO, with each pollutant included either as a confounder or modelled as an interaction term in separate analyses. Significant heterogeneity was observed in the case of PM_2.5_, NO_2_, and O_3_ (Table S5, Supporting Information).

First, only PM_2.5_ and O_3_ interaction model indicated a significant increase in the risk, and a substantial heterogeneity compared to single‐pollutant model. When NO_2_, SO_2_, and CO were added as confounders, effect of PM_2.5_ changed significantly. However, interaction models with these co‐pollutants indicated no significant modification, and the effect of PM_2.5_ became insignificant.

Similarly, NO_2_ consistently showed a significant association with OHCA across both interaction and confounder models when paired with PM_2.5_, SO_2_, and CO, with the overall model structure differing significantly from the single pollutant NO_2_ model. The effect size increased significantly in the interaction model suggesting strong modification. However, when paired with O_3_, the effect of NO_2_ loses significance, though the two‐pollutant models did not differ significantly from NO_2_‐only model.

In the case of O_3_, the relative risk dropped significantly when combined with the interaction of PM_2.5_, SO_2_, and CO, but when these pollutants were added as confounders, the effect of O_3_ remained stable. Relative risk per 1 µg m^−^^3^ increase of SO_2_ remained non‐significant across both types of two‐pollutant models. In contrast, CO showed a modest increase in risk when paired with O_3_ but lost its significance when analyzed with PM_2.5_, NO_2_, and SO_2_. None of the two‐pollutant models were significantly different from the CO‐only model.

## 4 Discussion

The association between short‐term exposure to air pollutants and OHCA events in the Lombardy region from 2016 to 2019 was investigated using a time‐stratified case‐crossover study design. While the relationship between OHCA incidents and pollution in a few areas of the region has been already studied,^[^ ^17^, ^46^, ^47^, ^48^ ^]^ this is the first study that analyzed the entire region while incorporating spatial heterogeneity in air pollution exposures using advanced air quality models and a relatively large sample size. We found that the risk of OHCA due to air pollution is not necessarily highest at lag 0 and undergoes effect modification across different sub‐groups and seasons, while also exhibiting synergistic interactions with co‐pollutants.

PM_2.5_, PM_10_, NO_2_, O_3_, and CO concentrations were positively associated with the OHCA risk for the overall population in the region. Lombardy, and especially its urban centers, witnesses numerous days a year surpassing the daily permissible limits for PM_2.5_, PM_10_, O_3_, and NO_2_, and their effects are coherently reflected in our findings with immediate risk at lag 0 and lag 1 for PM_2.5_, PM_10_ and O_3_, and from lag 2 to lag 5 for NO_2_. These results align with two meta‐analyses investigating OHCA risk linked to air pollution. Zhao et al.^[^ ^10^ ^]^ reported consistent association between OHCA and PM_2.5_, PM_10_, O_3_, and NO_2_, while finding no significant link with SO_2_ or CO across any lag period. Likewise, the second meta‐analysis examining the effect of air pollution on ambulance dispatches for cardiac arrests observed significant associations with PM_2.5_, CO and coarse particles from studies that included paramedic assessment.^[^ ^9^ ^]^

Comparison of our results with studies carried out in the same region revealed both consistencies and divergences. One study^[^ ^46^ ^]^ conducted on the southern and highly polluted provinces in the Po Valley in Lombardy found a positive association with OHCA for all pollutants, including SO_2_ (OR: 4.1; 95%CI: 2.2–7.8). SO_2_, however, did not exhibit meaningful association at the reasonable scale of 1 µg m^−^^3^ increase, largely due to the scarcity of data points exceeding 10 µg m^−^^3^. Notably, the exposure–response curve showed a significant effect only beyond 12 µg m^−^^3^, suggesting a potential threshold effect in SO_2_ related OHCA risk. While our study observed no significant effect of SO_2_ at the regional scale, stratification by rural districts showed a significant relative risk per 1 µg m^−^^3^ increase in SO_2_ (RR: 1.131; 95%CI: 1.003–1.275). The area under Gentile's study^[^ ^46^ ^]^ falls within our rural subset and supports the observed SO_2_ risk. Interestingly, the study also reported significant relative risks for all other air pollutants, with SO_2_ showing the highest risk. However, our findings identified SO_2_ as the only pollutant with a significant positive association in rural districts, albeit not statistically different from the urban counterparts. Our results on SO_2_ induced risk l of OHCA aligns with two other Japanese studies,^[^ ^19^, ^49^ ^]^ underscoring the importance of considering localized SO_2_ effects, even as broader literature often reports its association with OHCA as insignificant.

Similarly, Tobaldini et al.^[^ ^17^ ^]^ focused on the highly urbanized area of metropolitan Milan, applying DLNM study design to assess the effects of PM_10_ and temperature on OHCA, and reported significant associations at lag 3 (1.7%; 95%CI: 0.5%–2.9%). Stafoggia et al.^[^ ^48^ ^]^ also reported a steep increase in all‐cause mortality due to the joined effect of high PM_10_ levels (lag 0–1) and temperature in Milan. In agreement, we also report a significant effect of PM_2.5_ with a higher relative risk observed in urban districts, though at earlier lags of day 0 and lag 1 (RR: 1.035; 95%CI: 1.008–1.062) (comparative summary in Tables S6 and S7, Supporting Information).

In addition, subgroup analyses based on age and sex revealed an ncrease in relative risks, but the effect modification was not statistically significant. This contradicts results from previous studies that broadly reported higher effect sizes among males and older individuals.^[^ ^10^, ^14^, ^19^, ^47^ ^]^ Interestingly, some negative significant associations (protective effects) were observed for longer lags, predominantly in O_3_ (lag 5 and lag 6) among senior population. This result could be partly explained by the “harvesting effect” or “short‐term displacement,”^[^ ^50^ ^]^ which hypothesizes that acute exposure events have such a significant impact on vulnerable populations that they bring forward a portion of events that would have naturally occurred anyway, thus generating an under‐estimation of correlation at later lags. In this case too, many individuals with comorbidities, already nearing hospitalization, may have suffered a cardiac arrest due to increased pollution, leading to earlier hospitalization and lower incidence than the a‐priori likelihood at later lags. Bhaskaran et al.^[^ ^50^ ^]^ suggest that in such cases, assessing the cumulative association between exposure and outcome can provide a clearer picture of the true risk. This is illustrated in the exposure‐response curve of O_3_, where we used a 2 day running mean of pollution levels and observed that overall risk increases linearly as pollution concentration rises, especially among male sub‐group and urban districts.

Models stratified by seasons also demonstrated significant effect modifications for PM_2.5_ and O_3_, where Spring and Summer seasons were primarily linked to higher risk of OHCA, whereas Winter and Autumn showed no clear association despite having the highest number of OHCA. The existing literature on seasonal trends of OHCA is consistent with our study.^[^ ^51^ ^]^ However, limited studies have investigated the OHCA risk through sub‐group stratification. A meta‐analysis observed positive associations for SO_2_, NO_2_, and O_3_ in the warm season as well as for O_3_ in the cold season.^[^ ^10^ ^]^ Other studies also reported the association between OHCA and PM_10_ with increasing temperature,^[^ ^15^, ^17^ ^]^ PM_2.5_ and O_3_ in the warm season,^[^ ^20^, ^52^ ^]^ as well as O_3_ during winter (when its concentration levels are lower).^[^ ^13^, ^53^ ^]^ Despite the high concentration of pollutants in Winter and Autumn, a high number of OHCA seems more associated with temperature and other factors, such as season‐specific lifestyle changes. In contrast, the significant association of pollutants in Summer and Spring, despite their low concentrations, may be due to interactions with higher temperatures during these months.

The results from bi‐pollutant models showed insightful interaction and confounding effects between PM_2.5_, NO_2_, and O_3_. The inverse correlation between NO_2_ and O_3_ (Figure 2), reflecting the photochemical conversion of nitrogen oxides (NO_x_) into O_3_ under sunlight, is evident in their interaction models, where they lose their significant effect. This suggested multicollinearity and possible modeling bias due to overlapping variance. Moreover, NO_2_, having the largest effect on OHCA in single‐pollutant models, showed further increase in its effect size when interacted with PM_2.5_, SO_2_, and O_3_, indicating synergetic effect modification.

PM_2.5_ also demonstrated significant effect modification when paired with O_3_, resulting in elevated risk. This may be attributed to ozone's capability to react with particulate surfaces and aggravating its biological reactivity, a mechanism supported by experimental studies in both humans and animals.^[^ ^54^ ^]^ PM_2.5_ also showed significant changes while maintaining statistical significance when co‐pollutants were included as confounders. In contrast to NO_2_, which modified the effect of PM_2.5_, pollutants such as O_3_, SO_2_, and CO appear to improve model adjustment by accounting for shared exposure patterns and isolating the independent effect of PM_2.5_.^[^ ^13^, ^54^ ^]^ The bi‐pollutant analyses, therefore, underscore that the combined effect of air pollutants is not necessarily additive, and the decision to study them as effect modifier or as confounders should be guided by their temporal correlations, seasonal patterns, and overlapping pathophysiological mechanisms.

Furthermore, when we compared the results from the spatiotemporal model with the aggregated regional model, we observed that the effect sizes were not statistically different. Conversely, the effect sizes in terms of lags and sub‐group modifications were different between the two model types, suggesting the need to account for spatial heterogeneity in the risk assessment, as supported by a few studies that also produced a similar model on different cities or urban‐rural districts.^[^ ^8^, ^13^, ^45^ ^]^

A key strength of this study is its large scale, with 37613 cases across a population of 10.06 million exposed to unusually high pollution levels for a high‐income region. The findings in this study emphasize an urgent public health concern, particularly considering the steady rise in average and extreme temperature due to climate change. The interaction between heat and air pollution further amplifies acute cardiovascular risks, especially in urban areas.^[^ ^48^, ^55^ ^]^ Moreover, the significant effect modification between particulate matter and ozone, with elevated risks during the summer season, highlights a critical challenge for public health planning. While other environmental factors such as noise pollution, access to green areas, and socio‐economic disparities have been shown to influence OHCA risk,^[^ ^4^, ^56^ ^]^ they remain beyond the scope of the present analysis. Nonetheless, Italy's rapidly ageing population and increasing socio‐economic disparities reinforces the importance of localized risk assessments to support targeted public health strategies.^[^ ^57^ ^]^

Apart from the epidemiological insights, the study is grounded in a robust modeling framework that was rigorously parameterized though sensitivity analyses which evaluated alternative lag structures, and transformation functions of both primary exposures and meteorological confounders (S.4, Supporting Information). In terms of exposure measurement, our methodology addresses a common limitation in prior OHCA studies, which often relied solely upon air pollution data from ground monitoring stations, which can obscure spatial heterogeneity and lead to interpolation inaccuracies and missing data. Conversely, we used satellite‐based data that avoided such issues and provided temporally continuous coverage. Despite the low spatial resolution, averaging these data over the typically large district size can be considered adequate for district‐level analysis. Previous environmental epidemiologic studies carried out by our group on this territory,^[^ ^58^ ^]^ although on a different topic, showed that an increase in the granularity of the analysis is only feasible through spatial filtering, whose effect on the DLNM is uncertain. Furthermore, OHCA events have an average incidence of 1/1000 people per year: the chosen level of aggregation is therefore necessary to have enough cases to allow a short‐term impact analysis. Although this study reinforces the link between air pollution exposure and increased OHCA risk, we argue that certain inconsistencies in our effect estimates compared to previous studies are primarily due to differences in study context, population profiles, and median exposure levels. Moreover, epidemiological studies are inherently prone to inaccuracies arising from the temporal and spatial aggregation of exposure and health data, which can amplify errors.

Other limitations include data quality, as incidents were not classified by the International Classification of Diseases (ICD) standards, and clinical causes of OHCA were not identified, since different pollutants have been found to trigger OHCA via different cardiac mechanisms. For example, comorbidities (especially inherited cardiac arrhythmias, cardiomyopathies, and coronary artery anomalies) are important risk factors for sudden cardiac death amongst younger populations, possibly explaining our observation of high OHCA risk in this group due to particulate matter exposure.^[^ ^59^ ^]^ This absence of comorbidity data may have led to an underestimation of their confounding effects in some cases.

## 5 Conclusions

This large‐scale regional study demonstrates a spatiotemporal approach in evaluating OHCA risk from short‐term exposure to air pollution. This study adds to the growing body of epidemiological research that employs spatially and temporally continuous pollution concentration data as exposure metrics. However, challenges of downscaling exposure data persist, underscoring the need for improved methodologies to enhance precision.

Our findings revealed notable differences in the OHCA risks associated with different air pollutants, highlighting their distinct temporal pattern through delayed effect as well as their spatial variability between urban and rural areas. Moreover, despite not peaking during Summer and Spring, the pollutants exhibited significant associations during these seasons. These insights could be crucial to the local EMS to reorganize efforts during periods of heightened risk and allocate resources based on spatial risk patterns.

Furthermore, this study contributes to the limited research on the impact of air pollution on OHCA risk, especially for O_3_, SO_2_, and CO; their associated risks remain under debate. Future studies could expand on this work by incorporating additional stratification by socio‐economic status and lifestyle factors of OHCA patients.

## Conflict of Interest

The authors declare no conflict of interest.

## Supporting information



Supporting Information

## Data Availability

Data and code are available through GitHub repository: https://github.com/amruta‐mahakalkar/DLNM_2_stage_lomb.
