# Supplementary material for: Short‐Term Effect of Air Pollution on Out of Hospital Cardiac Arrest (OHCA) in Lombardy—A Case‐Crossover Spatiotemporal Study
Source: Glob Chall. 2025 Sep 29;9(11):e00241. doi: 10.1002/gch2.202500241 (PMC12602458; doi:10.1002/gch2.202500241)
Supplement: Supplementary file 1 — Supporting Information [file GCH2-9-e00241-s001.docx]

Supplementary Materials

**Short-term effect of air pollution on Out of Hospital Cardiac Arrest (OHCA) in Lombardy – a case-crossover spatiotemporal study**

**Contents**

**S.1.** Health data pre-processing

**S.2.** Results from exposure-lag-response assessment

**S.3.** Literature review summary

**S.4.** Sensitivity analysis

**Supplementary figures**

**Figure S.1:** OHCA case selection process

**Figure S.2.** Exposure–response curve: total and location-stratified effect of pollutants on OHCA.

**Figure S.3.** Exposure–response curve: total and age-stratified effect of pollutants on OHCA.

**Figure S.4.** Exposure–response curve: total and sex-stratified effect of pollutants on OHCA.

**Figure S.5.** Lag-response relationship between air pollution and OHCA by the regional model stratified by urban and rural setting, age and sex.

**Figure S.6.** Lag-response relationship between air pollution and OHCA with a co-pollutant included as an interaction term and as a confounder.

**Figure S.7.** Sensitivity analyses for individual relative risks of OHCA linked to air pollution at lag 0-3, lag 0-7, lag 0-10 and lag 0-14 days.

**Figure S.8.** Sensitivity analyses for cumulative relative risks of OHCA linked to air pollution at lag 0-3, lag 0-7, lag 0-10 and lag 0-14 days.

**Figure S.9.** Sensitivity analyses for relative risks of OHCA linked to air pollution by changing the smoothing functions and degree of freedom of predictor (pollutant) and its lag structure up to lag day 7.

**Figure S.10.** Sensitivity analyses for relative risks of OHCA linked to air pollution by changing the knot placement on the natural spline function of temperature and its lag structure up to lag 14.

**Figure S.11.** Sensitivity analyses for relative risks of OHCA linked to air pollution by changing the length of the running mean of the relative humidity and the degree of freedom of the natural splines.

**Supplementary tables**

**Table S.1.** Lag-response relationship between air pollutants and OHCA (main model)

**Table S.2.** Lag-response relationship between air pollutants and OHCA stratified by locational setting.

**Table S.3.** Lag-response relationship between air pollutants and OHCA stratified by seasons.

**Table S.4.** Lag-response relationship between air pollutants and OHCA stratified by age and sex.

**Table S.5.** Lag-response relationship between air pollutants and OHCA with a co-pollutant included as an interaction term and as a confounder.

**Table S.6.** Case studies on association between air pollution and OHCA.

**Table S.7.** Meta-analyses on association between air pollution and OHCA.

**Table S.8.** Comparative performance of models with varying lag length of the pollutants

**Table S.9.** Comparative performance of models with combinations of smoothing functions and lag structures of air pollutants

**Table S.10.** Comparative performance of models with varying temporal adjustment structures

S.1. Health data pre-processing

When experiencing a medical emergency in Lombardy, the public can dial the national emergency number 112 to receive urgent medical assistance. In the event of a reported out-of-hospital cardiac arrest (OHCA), the system immediately dispatches a medical vehicle with a team of trained healthcare professionals. The dataset includes the approximate coordinates of the telephonic calls seeking medical support in response to OHCA events and the corresponding call times.

Cardiac arrests are identified by the EMS personnel, and the outcomes are categorised as deceased, hospital admission or return of spontaneous circulation (ROSC). Cardiac arrest cases caused by trauma (majorly falls and road accidents) were excluded. Additionally, since cardiac arrest is confirmed and recorded only after the medical team arrives at the patient’s location, delays in response time could cause a cardiac arrest to develop from an initially non-cardiac condition. Therefore, to eliminate the confounding effect of delayed response time and ensure cohort homogeneity in the statistical model, 1916 (the top 4.85^th^ percentile) cases with response time exceeding 20 minutes were excluded.

**Figure S.1:** OHCA case selection process

All OHCA records from 2015 to 2019

**46,343**

Duplicate records

**853**

Records with invalid or missing information about patients

**2866**

Cases with response time over 20 min

**1916**

Cases due to external trauma

**3088**

Cases outside the region

**7**

OHCA cases used in the study

**37,613**

*Data cleaning*

*Data filtering*

S.2. Results from exposure-lag-response assessment

Table S.1. Association between air pollutants and OHCA (main model **^a^**) adjusted for temperature and relative humidity, by lag (days). Results are expressed as Relative Risk (RR) and 95% Confidence Intervals (95% CI), per 10 µg/m³ increase in PM₂.₅, PM₁₀, NO₂, O₃; 100 µg/m³ increase in CO; and 1 µg/m³ increase in SO₂.

| Lag (day) | RR | 95% CI |  | RR | 95% CI | RR | 95% CI |
| --- | --- | --- | --- | --- | --- | --- | --- |
|  | PM₂.₅ | |  | PM 10 | | NO₂ | |
| 0 | **1.0293** | **1.0062 - 1.0529** |  | **1.0255** | **1.0051 - 1.0462** | 0.9550 | 0.8954 - 1.0186 |
| 1 | **1.0136** | **1.0005 - 1.0270** |  | 1.0113 | 0.9996 - 1.0232 | 1.0002 | 0.9638 - 1.0380 |
| 2 | 1.0004 | 0.9904 - 1.0104 |  | 0.9994 | 0.9904 - 1.0085 | **1.0401** | **1.0070 - 1.0743** |
| 3 | 0.9916 | 0.9794 - 1.0040 |  | 0.9917 | 0.9807 - 1.0028 | **1.0664** | **1.0237 - 1.1108** |
| 4 | 0.9892 | 0.9767 - 1.0018 |  | 0.9900 | 0.9790 - 1.0012 | **1.0705** | **1.0285 - 1.1142** |
| 5 | 0.9931 | 0.9829 - 1.0035 |  | 0.9944 | 0.9853 - 1.0036 | **1.0523** | **1.0203 - 1.0854** |
| 6 | 1.0014 | 0.9893 - 1.0137 |  | 1.0030 | 0.9918 - 1.0143 | 1.0199 | 0.9805 - 1.0608 |
| 7 | 1.0120 | 0.9912 - 1.0332 |  | 1.0137 | 0.9945 - 1.0332 | 0.9814 | 0.9147 - 1.0529 |
|  | O₃ | |  | SO₂ | | CO | |
| 0 | 0.9986 | 0.9857 - 1.0116 |  | 1.0150 | 0.9450 - 1.0902 | 0.9919 | 0.9798 - 1.0041 |
| 1 | **1.0086** | **1.0011 - 1.0160** |  | 1.0206 | 0.9794 - 1.0637 | 0.9974 | 0.9906 - 1.0041 |
| 2 | 1.0063 | 0.9992 - 1.0134 |  | 1.0242 | 0.9904 - 1.0591 | 1.0021 | 0.9972 - 1.0071 |
| 3 | 0.9998 | 0.9949 - 1.0048 |  | 1.0235 | 0.9816 - 1.0672 | 1.0055 | 0.9990 - 1.0119 |
| 4 | 0.9965 | 0.9913 - 1.0016 |  | 1.0166 | 0.9748 - 1.0602 | **1.0067** | **1.0002 - 1.0132** |
| 5 | 0.9958 | 0.9916 - 1.0001 |  | 1.0036 | 0.9703 - 1.0381 | **1.0058** | **1.0008 - 1.0107** |
| 6 | 0.9969 | 0.9925 - 1.0014 |  | 0.9867 | 0.9474 - 1.0277 | 1.0034 | 0.9974 - 1.0094 |
| 7 | 0.9990 | 0.9911 - 1.0070 |  | 0.9681 | 0.9026 - 1.0384 | 1.0003 | 0.9894 - 1.0114 |

*^a^ Statistically significant results at p-value < 0.05 in bold.*

Figure S.2: Exposure–response curve showing the total and location-stratified effect of pollutants on OHCA adjusted for temperature and relative humidity. Solid lines represent relative risk (RR), and hatched or shaded areas represent 95% confidence intervals.


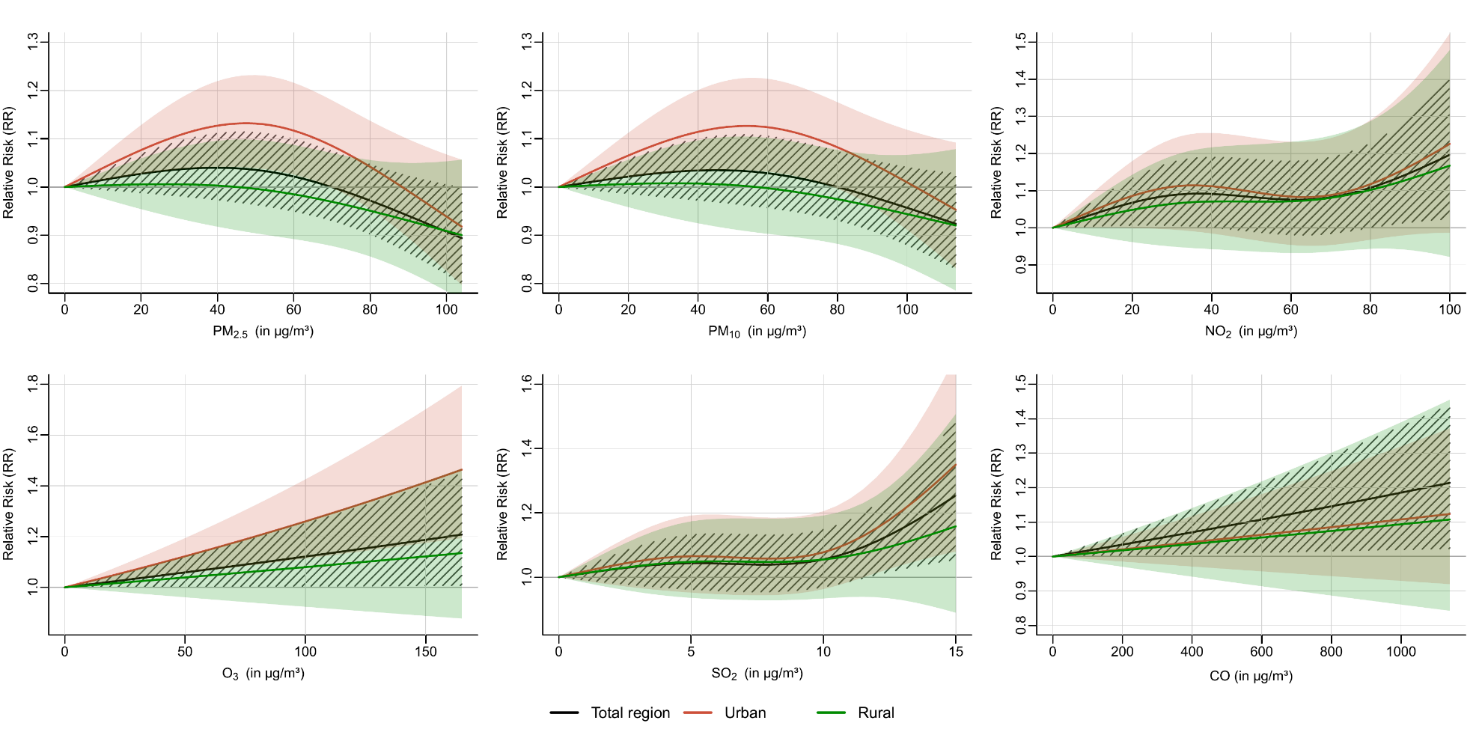


Figure S.3: Exposure–response curve showing the total and age-stratified effect of pollutants on OHCA adjusted for temperature and relative humidity. Solid lines represent relative risk (RR), and hatched or shaded areas represent 95% confidence intervals.


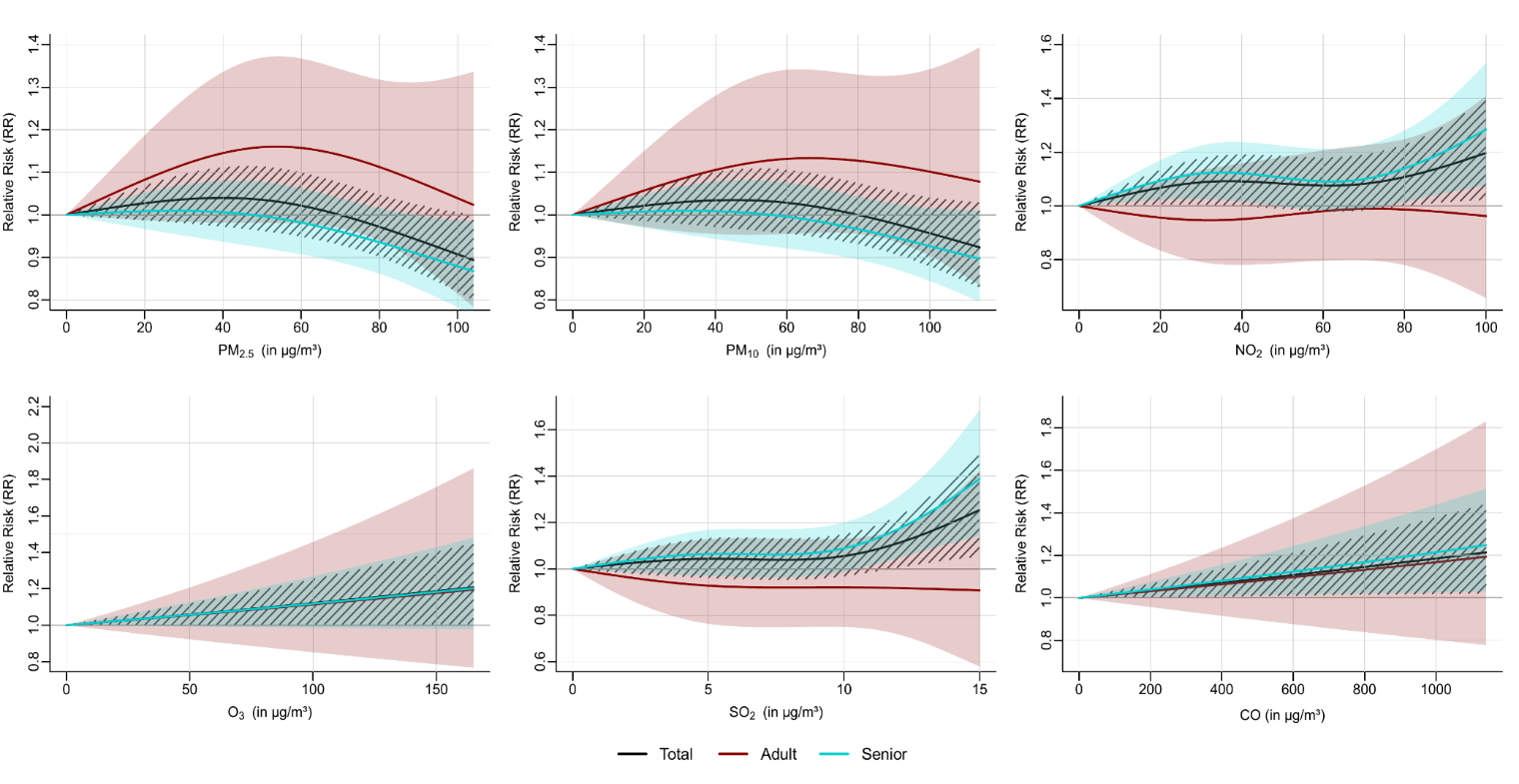


Figure S.4: Exposure–response curve showing the total and sex-stratified effect of pollutants on OHCA adjusted for temperature and relative humidity. Solid lines represent relative risk (RR), and hatched or shaded areas represent 95% confidence intervals.


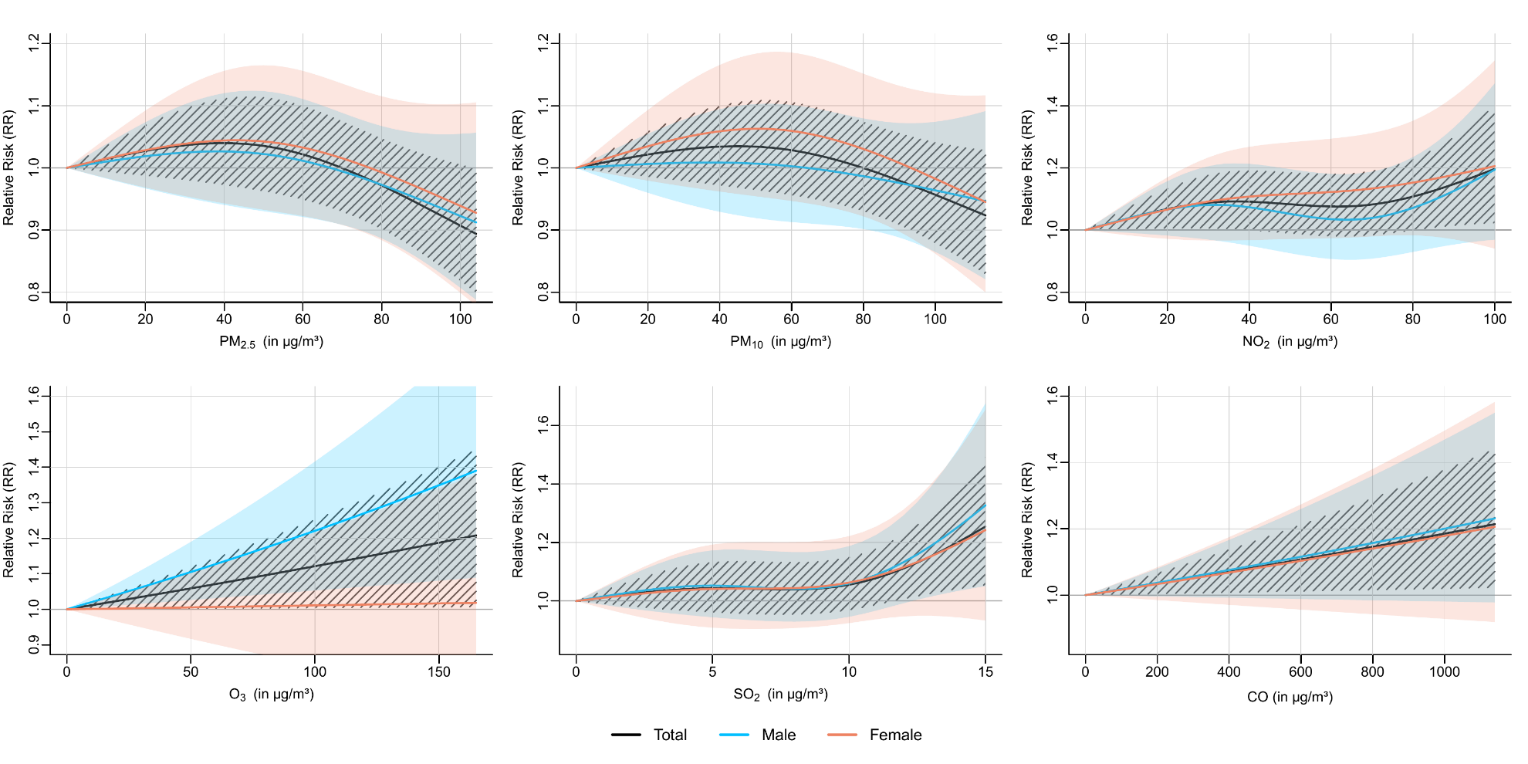


Table S.2. Association between air pollutants and OHCA adjusted for temperature and relative humidity, by lag (days), stratified by urban and rural setting **^a^**. Results are expressed as Relative Risk (RR) and 95% Confidence Intervals (95% CI), per 10 µg/m³ increase in PM₂.₅, PM₁₀, NO₂, O₃; 100 µg/m³ increase in CO; and 1 µg/m³ increase in SO₂.

| Lag (day) | RR | 95% CI |  | RR | 95% CI |  | RR | 95% CI |  | RR | 95% CI |
| --- | --- | --- | --- | --- | --- | --- | --- | --- | --- | --- | --- |
|  | PM₂.₅ - Urban | |  | PM₁₀ - Urban | |  | PM₂.₅ - Rural | |  | PM₁₀ - Rural | |
| 0 | **1.040** | **1.010 - 1.072** |  | **1.035** | **1.008 - 1.062** |  | 1.027 | 0.996 - 1.059 |  | 1.026 | 0.999 - 1.054 |
| 1 | **1.021** | **1.004 - 1.038** |  | **1.017** | **1.003 - 1.032** |  | 1.014 | 0.997 - 1.031 |  | 1.013 | 0.998 - 1.029 |
| 2 | 1.004 | 0.993 - 1.016 |  | 1.003 | 0.992 - 1.013 |  | 1.002 | 0.989 - 1.015 |  | 1.002 | 0.990 - 1.014 |
| 3 | 0.993 | 0.978 - 1.008 |  | 0.993 | 0.980 - 1.006 |  | 0.993 | 0.976 - 1.010 |  | 0.994 | 0.979 - 1.009 |
| 4 | 0.989 | 0.974 - 1.005 |  | 0.990 | 0.977 - 1.004 |  | 0.988 | 0.971 - 1.005 |  | 0.989 | 0.974 - 1.005 |
| 5 | 0.993 | 0.981 - 1.006 |  | 0.995 | 0.983 - 1.006 |  | 0.987 | 0.973 - 1.000 |  | 0.988 | 0.976 - 1.001 |
| 6 | 1.002 | 0.987 - 1.018 |  | 1.004 | 0.989 - 1.018 |  | 0.988 | 0.971 - 1.005 |  | 0.990 | 0.975 - 1.006 |
| 7 | 1.014 | 0.987 - 1.042 |  | 1.016 | 0.991 - 1.041 |  | 0.991 | 0.961 - 1.021 |  | 0.993 | 0.967 - 1.021 |
|  | NO₂ - Urban | |  | O₃ - Urban | |  | NO₂ - Rural | |  | O₃ - Rural | |
| 0 | 0.934 | 0.865 - 1.009 |  | 0.998 | 0.984 - 1.013 |  | 1.004 | 0.893 - 1.129 |  | 1.003 | 0.984 - 1.022 |
| 1 | 0.986 | 0.943 - 1.030 |  | **1.010** | **1.002 - 1.019** |  | 1.032 | 0.965 - 1.103 |  | 1.008 | 0.998 - 1.019 |
| 2 | 1.032 | 0.993 - 1.071 |  | **1.011** | **1.003 - 1.018** |  | 1.055 | 0.992 - 1.121 |  | 1.004 | 0.995 - 1.014 |
| 3 | **1.063** | **1.014 - 1.115** |  | **1.006** | **1.001 - 1.011** |  | 1.067 | 0.987 - 1.154 |  | 0.997 | 0.991 - 1.004 |
| 4 | **1.071** | **1.022 - 1.122** |  | 1.002 | 0.996 - 1.008 |  | 1.064 | 0.986 - 1.148 |  | 0.994 | 0.987 - 1.001 |
| 5 | **1.054** | **1.015 - 1.094** |  | 0.999 | 0.994 - 1.004 |  | 1.045 | 0.988 - 1.106 |  | **0.993** | **0.988 - 0.999** |
| 6 | 1.021 | 0.972 - 1.072 |  | 0.997 | 0.991 - 1.002 |  | 1.017 | 0.947 - 1.092 |  | 0.995 | 0.989 - 1.001 |
| 7 | 0.982 | 0.901 - 1.069 |  | 0.994 | 0.984 - 1.004 |  | 0.984 | 0.864 - 1.120 |  | 0.997 | 0.987 - 1.008 |
|  | SO₂ - Urban | |  | CO - Urban | |  | SO₂ - Rural | |  | CO - Rural | |
| 0 | 0.980 | 0.899 - 1.069 |  | 0.993 | 0.980 - 1.008 |  | **1.131** | **1.003 - 1.275** |  | 1.003 | 0.984 - 1.022 |
| 1 | 1.012 | 0.963 - 1.064 |  | 0.998 | 0.990 - 1.005 |  | **1.077** | **1.006 - 1.152** |  | 1.003 | 0.992 - 1.014 |
| 2 | 1.038 | 0.996 - 1.082 |  | 1.001 | 0.996 - 1.007 |  | 1.031 | 0.978 - 1.086 |  | 1.003 | 0.995 - 1.011 |
| 3 | 1.050 | 0.997 - 1.106 |  | 1.004 | 0.996 - 1.011 |  | 0.998 | 0.934 - 1.066 |  | 1.003 | 0.993 - 1.012 |
| 4 | 1.041 | 0.989 - 1.096 |  | 1.004 | 0.997 - 1.012 |  | 0.982 | 0.918 - 1.051 |  | 1.002 | 0.993 - 1.012 |
| 5 | 1.012 | 0.970 - 1.055 |  | 1.003 | 0.997 - 1.009 |  | 0.983 | 0.931 - 1.037 |  | 1.002 | 0.994 - 1.009 |
| 6 | 0.971 | 0.921 - 1.023 |  | 1.000 | 0.993 - 1.007 |  | 0.994 | 0.934 - 1.059 |  | 1.001 | 0.991 - 1.010 |
| 7 | 0.925 | 0.845 - 1.012 |  | 0.997 | 0.984 - 1.010 |  | 1.012 | 0.908 - 1.128 |  | 0.999 | 0.982 - 1.017 |

*^a^ Statistically significant results at p-value < 0.05 in bold.*

** Statistically significant difference (p-value < 0.05) of effect estimates between the sub-group and reference (urban) from z-test.*

*Note: No statistical difference was observed between effect estimates of main and stratified models by urban-rural setting.*

Table S.3. Association between air pollutants and OHCA adjusted for temperature and relative humidity, by lag (days), stratified by seasons **^a^**. Results are expressed as Relative Risk (RR) and 95% Confidence Intervals (95% CI), per 10 µg/m³ increase in PM₂.₅, PM₁₀, NO₂, O₃; 100 µg/m³ increase in CO; and 1 µg/m³ increase in SO₂.

| Lag (day) | RR | 95% CI |  | RR | 95% CI |  | RR | 95% CI |  | RR | 95% CI |
| --- | --- | --- | --- | --- | --- | --- | --- | --- | --- | --- | --- |
|  | PM₂.₅ – Summer | |  | PM₁₀ – Summer | |  | PM₂.₅ – Spring | |  | PM₁₀ – Spring | |
| 0 | 1.132 | 0.993 - 1.289 |  | 1.060 | 0.961 - 1.170 |  | 1.051 | 0.992 - 1.113 |  | 1.045 | 0.990 - 1.102 |
| 1 | **1.092** | **1.008 - 1.183*** |  | 1.043 | 0.980 - 1.110 |  | 1.029 | 0.991 - 1.068 |  | 1.026 | 0.990 - 1.063 |
| 2 | **1.059** | **1.001 - 1.120*** |  | 1.028 | 0.981 - 1.077 |  | 1.010 | 0.979 - 1.042 |  | 1.009 | 0.980 - 1.040 |
| 3 | 1.035 | 0.974 - 1.100 |  | 1.017 | 0.967 - 1.069 |  | 0.997 | 0.963 - 1.032 |  | 0.998 | 0.967 - 1.030 |
| 4 | 1.025 | 0.963 - 1.092 |  | 1.010 | 0.960 - 1.063 |  | 0.993 | 0.960 - 1.027 |  | 0.994 | 0.963 - 1.025 |
| 5 | 1.029 | 0.975 - 1.085 |  | 1.009 | 0.966 - 1.055 |  | 0.997 | 0.968 - 1.027 |  | 0.996 | 0.969 - 1.024 |
| 6 | 1.041 | 0.981 - 1.104 |  | 1.012 | 0.963 - 1.063 |  | 1.007 | 0.971 - 1.044 |  | 1.004 | 0.970 - 1.039 |
| 7 | 1.058 | 0.963 - 1.162 |  | 1.016 | 0.940 - 1.097 |  | 1.020 | 0.963 - 1.080 |  | 1.014 | 0.962 - 1.068 |
|  | NO₂ _–_ Summer | |  | O₃ – Summer | |  | NO₂ – Spring | |  | O₃ – Spring | |
| 0 | 1.177 | 0.830 - 1.671 |  | 1.009 | 0.980 - 1.038 |  | 0.932 | 0.781 - 1.112 |  | 0.983 | 0.958 - 1.009 |
| 1 | 1.070 | 0.862 - 1.329 |  | 1.011 | 0.996 - 1.026 |  | 0.949 | 0.848 - 1.062 |  | **1.022** | **1.007 - 1.038** |
| 2 | 0.986 | 0.823 - 1.181 |  | 1.006 | 0.991 - 1.020 |  | 0.962 | 0.870 - 1.064 |  | **1.020** | **1.006 - 1.034** |
| 3 | 0.933 | 0.754 - 1.154 |  | 0.999 | 0.989 - 1.010 |  | 0.968 | 0.860 - 1.089 |  | 1.002 | 0.993 - 1.012 |
| 4 | 0.918 | 0.742 - 1.136 |  | 0.996 | 0.985 - 1.008 |  | 0.961 | 0.856 - 1.080 |  | 0.993 | 0.983 - 1.004 |
| 5 | 0.940 | 0.784 - 1.126 |  | 0.996 | 0.986 - 1.006 |  | 0.943 | 0.855 - 1.041 |  | 0.992 | 0.983 - 1.001 |
| 6 | 0.988 | 0.800 - 1.220 |  | 0.997 | 0.987 - 1.008 |  | 0.918 | 0.818 - 1.031 |  | 0.996 | 0.988 - 1.005 |
| 7 | 1.053 | 0.749 - 1.480 |  | 1.000 | 0.982 - 1.018 |  | 0.890 | 0.739 - 1.072 |  | 1.003 | 0.988 - 1.019 |
|  | SO₂ _–_ Summer | |  | CO – Summer | |  | SO₂ – Spring | |  | CO – Spring | |
| 0 | 1.105 | 0.862 - 1.416 |  | 1.057 | 0.961 - 1.161 |  | 1.011 | 0.832 - 1.228 |  | 0.990 | 0.949 - 1.034 |
| 1 | 1.092 | 0.946 - 1.261 |  | 1.029 | 0.977 - 1.083 |  | 1.032 | 0.912 - 1.167 |  | 0.992 | 0.967 - 1.018 |
| 2 | 1.079 | 0.962 - 1.211 |  | 1.005 | 0.971 - 1.041 |  | 1.047 | 0.946 - 1.158 |  | 0.994 | 0.973 - 1.015 |
| 3 | 1.065 | 0.923 - 1.229 |  | 0.990 | 0.946 - 1.035 |  | 1.050 | 0.936 - 1.178 |  | 0.995 | 0.970 - 1.021 |
| 4 | 1.049 | 0.907 - 1.214 |  | 0.986 | 0.941 - 1.032 |  | 1.035 | 0.924 - 1.159 |  | 0.996 | 0.971 - 1.022 |
| 5 | 1.032 | 0.916 - 1.163 |  | 0.992 | 0.960 - 1.026 |  | 1.003 | 0.912 - 1.102 |  | 0.998 | 0.978 - 1.018 |
| 6 | 1.014 | 0.884 - 1.164 |  | 1.007 | 0.970 - 1.045 |  | 0.960 | 0.860 - 1.073 |  | 0.999 | 0.975 - 1.023 |
| 7 | 0.996 | 0.791 - 1.254 |  | 1.025 | 0.955 - 1.101 |  | 0.915 | 0.763 - 1.096 |  | 1.000 | 0.960 - 1.042 |
|  | PM₂.₅ – Autumn | |  | PM₁₀ – Autumn | |  | PM₂.₅ – Winter | |  | PM₁₀ – Winter | |
| 0 | 1.028 | 0.981 - 1.077 |  | 1.022 | 0.980 - 1.066 |  | 1.010 | 0.968 - 1.053 |  | 1.016 | 0.977 - 1.056 |
| 1 | 1.024 | 0.998 - 1.051 |  | 1.020 | 0.997 - 1.044 |  | 0.992 | 0.969 - 1.016 |  | 0.997 | 0.976 - 1.019 |
| 2 | 1.021 | 0.999 - 1.043 |  | 1.018 | 0.998 - 1.037 |  | 0.978 | 0.962 - 0.995 |  | 0.981 | 0.966 - 0.997 |
| 3 | 1.017 | 0.988 - 1.046 |  | 1.015 | 0.989 - 1.041 |  | 0.969 | 0.948 - 0.989 |  | 0.971 | 0.953 - 0.990 |
| 4 | 1.012 | 0.983 - 1.041 |  | 1.011 | 0.986 - 1.037 |  | 0.966 | 0.946 - 0.987 |  | 0.969 | 0.950 - 0.988 |
| 5 | 1.006 | 0.985 - 1.027 |  | 1.006 | 0.988 - 1.026 |  | 0.972 | 0.955 - 0.989 |  | 0.974 | 0.959 - 0.989 |
| 6 | 0.999 | 0.975 - 1.025 |  | 1.002 | 0.980 - 1.024 |  | 0.982 | 0.961 - 1.003 |  | 0.985 | 0.966 - 1.004 |
| 7 | 0.993 | 0.948 - 1.039 |  | 0.996 | 0.957 - 1.038 |  | 0.994 | 0.958 - 1.032 |  | 0.998 | 0.964 - 1.033 |
|  | NO₂ _–_ Autumn | |  | O₃ – Autumn | |  | NO₂ – Winter | |  | O₃ – Winter | |
| 0 | 1.012 | 0.887 - 1.155 |  | 1.007 | 0.975 - 1.040 |  | 1.007 | 0.923 - 1.099 |  | 1.001 | 0.971 - 1.031 |
| 1 | 1.037 | 0.957 - 1.124 |  | 0.991 | 0.973 - 1.011 |  | 0.986 | 0.935 - 1.040 |  | 1.015 | 0.998 - 1.032 |
| 2 | 1.057 | 0.986 - 1.134 |  | 0.992 | 0.975 - 1.010 |  | 0.968 | 0.928 - 1.010 |  | 1.010 | 0.996 - 1.024 |
| 3 | 1.065 | 0.979 - 1.159 |  | 0.999 | 0.987 - 1.012 |  | 0.957 | 0.911 - 1.006 |  | 0.999 | 0.989 - 1.009 |
| 4 | 1.056 | 0.971 - 1.149 |  | 1.004 | 0.991 - 1.018 |  | 0.956 | 0.910 - 1.005 |  | 0.995 | 0.984 - 1.006 |
| 5 | 1.031 | 0.962 - 1.104 |  | 1.007 | 0.996 - 1.018 |  | 0.965 | 0.925 - 1.006 |  | 0.996 | 0.986 - 1.005 |
| 6 | 0.995 | 0.918 - 1.077 |  | 1.009 | 0.997 - 1.020 |  | 0.980 | 0.932 - 1.030 |  | 1.000 | 0.990 - 1.009 |
| 7 | 0.954 | 0.836 - 1.089 |  | 1.009 | 0.989 - 1.030 |  | 0.998 | 0.921 - 1.082 |  | 1.005 | 0.989 - 1.022 |
|  | SO₂ _–_ Autumn | |  | CO – Autumn | |  | SO₂ – Winter | |  | CO – Winter | |
| 0 | 1.005 | 0.862 - 1.172 |  | 1.015 | 0.986 - 1.045 |  | 0.944 | 0.843 - 1.057 |  | 0.987 | 0.972 - 1.004 |
| 1 | 1.037 | 0.950 - 1.131 |  | 1.012 | 0.997 - 1.028 |  | 0.950 | 0.888 - 1.016 |  | 0.996 | 0.986 - 1.006 |
| 2 | 1.062 | 0.982 - 1.148 |  | 1.010 | 0.997 - 1.022 |  | 0.955 | 0.901 - 1.011 |  | 1.003 | 0.996 - 1.010 |
| 3 | 1.073 | 0.970 - 1.186 |  | 1.007 | 0.990 - 1.024 |  | 0.957 | 0.891 - 1.027 |  | **1.008** | **1.000 - 1.017** |
| 4 | 1.062 | 0.959 - 1.175 |  | 1.003 | 0.986 - 1.020 |  | 0.955 | 0.890 - 1.026 |  | **1.010** | **1.002 - 1.018** |
| 5 | 1.030 | 0.949 - 1.117 |  | 0.999 | 0.987 - 1.011 |  | 0.951 | 0.897 - 1.007 |  | **1.008** | **1.001 - 1.015** |
| 6 | 0.985 | 0.895 - 1.085 |  | 0.994 | 0.979 - 1.009 |  | 0.943 | 0.884 - 1.007 |  | 1.004 | 0.996 - 1.012 |
| 7 | 0.936 | 0.795 - 1.102 |  | 0.989 | 0.961 - 1.018 |  | 0.935 | 0.839 - 1.042 |  | 0.999 | 0.984 - 1.013 |

*^a^ Statistically significant results at p-value < 0.05 in bold.*

** Statistically significant difference (p-value < 0.05) of effect estimates between the sub-group and reference (total OHCA) from z-test.*

Table S.4. Association between air pollutants and OHCA adjusted for temperature and relative humidity, by lag (days), stratified by sex **^a^**. Results are expressed as Relative Risk (RR) and 95% Confidence Intervals (95% CI), per 10 µg/m³ increase in PM₂.₅, PM₁₀, NO₂, O₃; 100 µg/m³ increase in CO; and 1 µg/m³ increase in SO₂.

| Lag (day) | RR | 95% CI |  | RR | 95% CI |  | RR | 95% CI |  | RR | 95% CI |
| --- | --- | --- | --- | --- | --- | --- | --- | --- | --- | --- | --- |
|  | PM₂.₅ - Male | |  | PM₁₀- Male | |  | PM₂.₅- Female | |  | PM₁₀- Female | |
| 0 | 1.030 | 0.999 - 1.062 |  | 1.024 | 0.996 - 1.052 |  | 1.029 | 0.990 - 1.070 |  | 1.031 | 0.995 - 1.068 |
| 1 | 1.014 | 0.996 - 1.032 |  | 1.011 | 0.995 - 1.027 |  | 1.014 | 0.993 - 1.036 |  | 1.014 | 0.995 - 1.034 |
| 2 | 1.000 | 0.987 - 1.014 |  | 0.999 | 0.987 - 1.012 |  | 1.001 | 0.985 - 1.018 |  | 1.000 | 0.986 - 1.015 |
| 3 | 0.990 | 0.974 - 1.007 |  | 0.991 | 0.977 - 1.006 |  | 0.994 | 0.973 - 1.015 |  | 0.993 | 0.974 - 1.012 |
| 4 | 0.986 | 0.970 - 1.002 |  | 0.989 | 0.974 - 1.004 |  | 0.995 | 0.974 - 1.016 |  | 0.993 | 0.974 - 1.013 |
| 5 | 0.987 | 0.974 - 1.001 |  | 0.990 | 0.979 - 1.002 |  | 1.003 | 0.987 - 1.020 |  | 1.002 | 0.987 - 1.018 |
| 6 | 0.992 | 0.977 - 1.008 |  | 0.996 | 0.981 - 1.010 |  | 1.017 | 0.996 - 1.038 |  | 1.017 | 0.998 - 1.037 |
| 7 | 0.999 | 0.972 - 1.027 |  | 1.002 | 0.978 - 1.028 |  | 1.034 | 0.996 - 1.072 |  | **1.035** | **1.001 - 1.071** |
|  | NO₂ - Male | |  | O₃ - Male | |  | NO₂ - Female | |  | O₃ - Female | |
| 0 | 0.973 | 0.892 - 1.060 |  | 1.003 | 0.986 - 1.021 |  | 0.913 | 0.822 - 1.015 |  | 0.993 | 0.974 - 1.014 |
| 1 | 1.020 | 0.970 - 1.073 |  | **1.012** | **1.002 - 1.022** |  | 0.962 | 0.907 - 1.020 |  | 1.005 | 0.993 - 1.016 |
| 2 | **1.061** | **1.018 - 1.106** |  | 1.008 | 0.999 - 1.018 |  | 1.006 | 0.959 - 1.056 |  | 1.003 | 0.993 - 1.013 |
| 3 | **1.086** | **1.031 - 1.143** |  | 1.001 | 0.994 - 1.008 |  | 1.039 | 0.976 - 1.106 |  | 0.997 | 0.990 - 1.004 |
| 4 | **1.084** | **1.028 - 1.142** |  | 0.997 | 0.990 - 1.004 |  | 1.052 | 0.989 - 1.119 |  | 0.994 | 0.986 - 1.002 |
| 5 | **1.055** | **1.010 - 1.102** |  | 0.997 | 0.991 - 1.002 |  | 1.044 | 0.995 - 1.095 |  | 0.994 | 0.987 - 1.000 |
| 6 | 1.011 | 0.959 - 1.065 |  | 0.998 | 0.992 - 1.004 |  | 1.023 | 0.960 - 1.090 |  | 0.995 | 0.988 - 1.002 |
| 7 | 0.960 | 0.879 - 1.049 |  | 1.000 | 0.990 - 1.011 |  | 0.996 | 0.888 - 1.117 |  | 0.997 | 0.985 - 1.010 |
|  | SO₂ - Male | |  | CO - Male | |  | SO₂ - Female | |  | CO - Female | |
| 0 | 1.002 | 0.911 - 1.103 |  | 0.999 | 0.983 - 1.014 |  | 1.009 | 0.900 - 1.131 |  | 0.9839 | 0.9652 - 1.0030 |
| 1 | 1.016 | 0.961 - 1.074 |  | 1.002 | 0.993 - 1.011 |  | 1.012 | 0.947 - 1.081 |  | 0.9925 | 0.9822 - 1.0029 |
| 2 | 1.026 | 0.981 - 1.074 |  | 1.005 | 0.998 - 1.011 |  | 1.012 | 0.958 - 1.068 |  | 1.0001 | 0.9922 - 1.0080 |
| 3 | 1.031 | 0.975 - 1.091 |  | 1.006 | 0.998 - 1.015 |  | 1.008 | 0.942 - 1.078 |  | 1.0056 | 0.9952 - 1.0161 |
| 4 | 1.028 | 0.972 - 1.088 |  | 1.007 | 0.998 - 1.015 |  | 0.997 | 0.932 - 1.067 |  | 1.0080 | 0.9976 - 1.0185 |
| 5 | 1.017 | 0.971 - 1.064 |  | 1.005 | 0.999 - 1.012 |  | 0.981 | 0.928 - 1.036 |  | 1.0073 | 0.9996 - 1.0150 |
| 6 | 0.999 | 0.944 - 1.058 |  | 1.003 | 0.995 - 1.011 |  | 0.960 | 0.897 - 1.027 |  | 1.0045 | 0.9947 - 1.0144 |
| 7 | 0.980 | 0.889 - 1.081 |  | 1.001 | 0.986 - 1.015 |  | 0.938 | 0.835 - 1.053 |  | 1.0006 | 0.9822 - 1.0192 |
|  |  |  |  |  |  |  |  |  |  |  |  |
|  | PM₂.₅ - Age ≤ 65 | |  | PM₁₀- Age ≤ 65 | |  | PM₂.₅ - Age > 65 | |  | PM₁₀ - Age > 65 | |
| 0 | **1.070** | **1.007 - 1.136** |  | 1.051 | 0.998 - 1.107 |  | 1.020 | 0.993 - 1.048 |  | 1.020 | 0.996 - 1.044 |
| 1 | **1.043** | **1.007 - 1.081** |  | **1.034** | **1.003 - 1.065** |  | 1.006 | 0.991 - 1.022 |  | 1.006 | 0.992 - 1.020 |
| 2 | 1.020 | 0.994 - 1.047 |  | 1.018 | 0.995 - 1.042 |  | 0.995 | 0.984 - 1.007 |  | 0.995 | 0.984 - 1.005 |
| 3 | 1.002 | 0.971 - 1.034 |  | 1.005 | 0.978 - 1.034 |  | 0.989 | 0.975 - 1.003 |  | 0.988 | 0.976 - 1.001 |
| 4 | 0.990 | 0.960 - 1.022 |  | 0.997 | 0.969 - 1.025 |  | 0.989 | 0.974 - 1.003 |  | 0.989 | 0.976 - 1.002 |
| 5 | 0.985 | 0.960 - 1.011 |  | 0.992 | 0.970 - 1.015 |  | 0.995 | 0.984 - 1.007 |  | 0.996 | 0.986 - 1.006 |
| 6 | 0.985 | 0.955 - 1.015 |  | 0.990 | 0.963 - 1.017 |  | 1.007 | 0.993 - 1.021 |  | 1.008 | 0.996 - 1.021 |
| 7 | 0.986 | 0.937 - 1.038 |  | 0.989 | 0.944 - 1.035 |  | 1.021 | 0.996 - 1.046 |  | **1.023** | **1.001 - 1.046** |
|  | NO₂ - Age ≤ 65 | |  | O₃ - Age ≤ 65 | |  | NO₂ - Age > 65 | |  | O₃ - Age > 65 | |
| 0 | 0.924 | 0.793 - 1.077 |  | 1.002 | 0.971 - 1.033 |  | 0.962 | 0.894 - 1.036 |  | 0.997 | 0.983 - 1.012 |
| 1 | 0.959 | 0.877 - 1.049 |  | 1.011 | 0.994 - 1.028 |  | 1.006 | 0.965 - 1.049 |  | 1.008 | 0.999 - 1.016 |
| 2 | 0.992 | 0.918 - 1.072 |  | 1.006 | 0.991 - 1.022 |  | **1.045** | **1.008 - 1.082** |  | 1.006 | 0.998 - 1.014 |
| 3 | 1.017 | 0.922 - 1.121 |  | 0.999 | 0.986 - 1.011 |  | **1.069** | **1.023 - 1.119** |  | 1.000 | 0.994 - 1.006 |
| 4 | 1.030 | 0.933 - 1.138 |  | 0.997 | 0.984 - 1.011 |  | **1.072** | **1.026 - 1.121** |  | 0.996 | 0.990 - 1.002 |
| 5 | 1.031 | 0.951 - 1.119 |  | 1.001 | 0.990 - 1.012 |  | **1.053** | **1.017 - 1.090** |  | **0.995** | **0.990 - 0.999** |
| 6 | 1.024 | 0.933 - 1.124 |  | 1.009 | 0.998 - 1.019 |  | 1.019 | 0.975 - 1.066 |  | **0.994** | **0.989 - 1.000** |
| 7 | 1.012 | 0.868 - 1.181 |  | 1.018 | 0.999 - 1.038 |  | 0.980 | 0.905 - 1.060 |  | 0.995 | 0.986 - 1.004 |
|  | SO₂ - Age ≤ 65 | |  | CO - Age ≤ 65 | |  | SO₂ - Age > 65 | |  | CO - Age > 65 | |
| 0 | 1.113 | 0.926 - 1.337 |  | 0.984 | 0.956 - 1.013 |  | 0.982 | 0.906 - 1.064 |  | 0.992 | 0.979 - 1.005 |
| 1 | 1.032 | 0.928 - 1.148 |  | 0.994 | 0.978 - 1.011 |  | 1.010 | 0.964 - 1.058 |  | 0.997 | 0.990 - 1.005 |
| 2 | 0.969 | 0.885 - 1.061 |  | 1.003 | 0.990 - 1.015 |  | 1.032 | 0.994 - 1.073 |  | 1.002 | 0.997 - 1.008 |
| 3 | 0.931 | 0.831 - 1.043 |  | 1.008 | 0.993 - 1.024 |  | 1.043 | 0.995 - 1.094 |  | 1.006 | 0.999 - 1.013 |
| 4 | 0.927 | 0.828 - 1.037 |  | 1.009 | 0.993 - 1.024 |  | 1.036 | 0.988 - 1.087 |  | **1.008** | **1.0003 - 1.015** |
| 5 | 0.955 | 0.874 - 1.043 |  | 1.004 | 0.992 - 1.016 |  | 1.011 | 0.973 - 1.051 |  | **1.007** | **1.002 - 1.013** |
| 6 | 1.008 | 0.906 - 1.122 |  | 0.997 | 0.982 - 1.012 |  | 0.976 | 0.931 - 1.022 |  | 1.005 | 0.998 - 1.012 |
| 7 | 1.077 | 0.893 - 1.298 |  | 0.988 | 0.961 - 1.014 |  | 0.936 | 0.864 - 1.013 |  | 1.003 | 0.990 - 1.015 |

*^a^ Statistically significant results at p-value < 0.05 in bold.*

** Statistically significant difference (p-value < 0.05) of effect estimates between the sub-group and reference and references (male and age ≤ 65) from z-test.*

*Note: No statistical difference was observed between effect estimates of main and age or sex stratified models.*

Figure S.5: Association of OHCA to pollutants in the regional model stratified by urban and rural setting (plot above), age and sex (plot below), adjusted for temperature and relative humidity, by lag (days), and expressed in Relative Risk (RR) and 95% Confidence Intervals (95% CI), per 10 µg/m³ increase in PM₂.₅, PM₁₀, NO₂, O₃; per 100 µg/m³ increase in CO; and per 1 µg/m³ increase in SO₂.


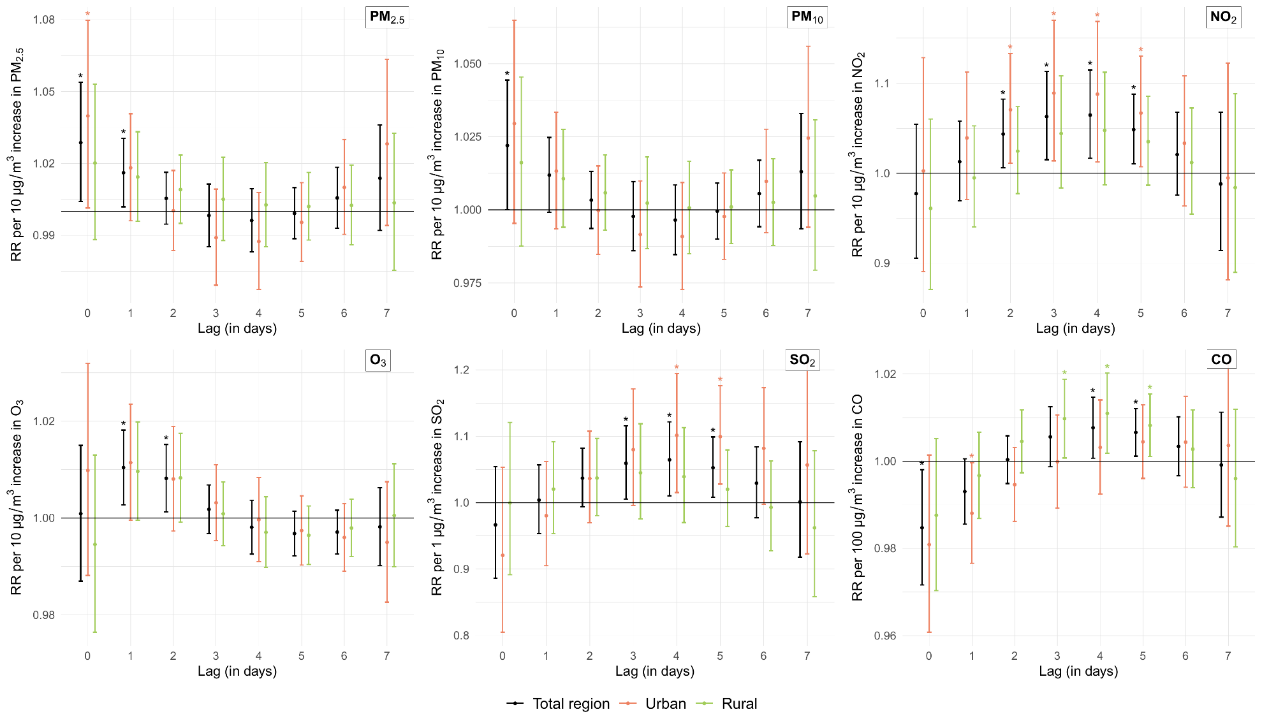


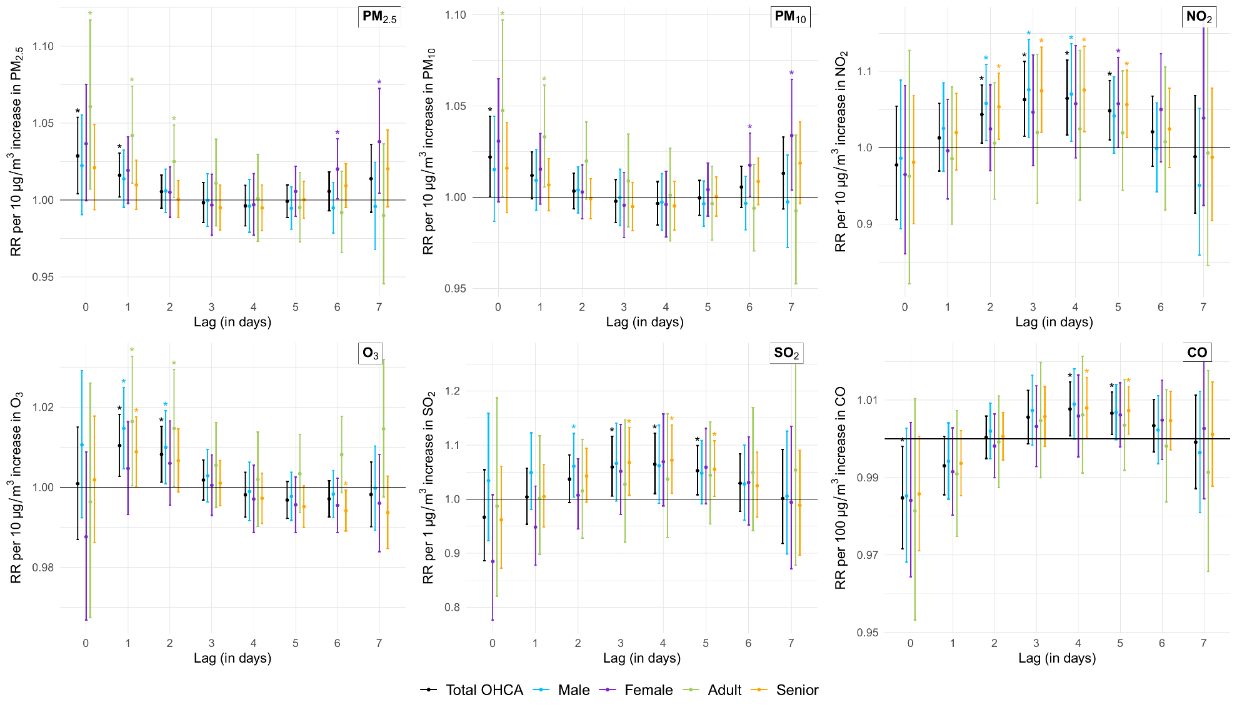


** Statistically significant results at p-value < 0.05 in bold.*

*** Statistically significant difference (p-value < 0.05) of effect estimates between the sub-group and its counterpart from the spatiotemporal model, based on z-test.*

*Note: No statistical difference was observed between effect estimates of regional and spatiotemporal models.*

Table S.5. Association between air pollutants and OHCA with a co-pollutant included as an interaction term and as a confounder, by lag (days), adjusted for temperature and relative humidity. Results are expressed as Relative Risk (RR)^a^ and 95% Confidence Intervals (95% CI), per 10 µg/m³ increase in PM₂.₅, PM₁₀, NO₂, O₃; 100 µg/m³ increase in CO; and 1 µg/m³ increase in SO₂.

| Pollutant | Co-Pollutant | Interaction | p-value* | Confounder | p-value |
| --- | --- | --- | --- | --- | --- |
| PM₂.₅ | **1.029 (1.006 - 1.053)^b^** | | | | |
| PM₂.₅ | NO₂ | 1.006 (0.960 - 1.054) | 0.191 | **1.029 (1.000 - 1.060)*** | 0.008 |
| PM₂.₅ | O₃ | **1.034 (1.000 - 1.069)*** | 0.013 | **1.025 (1.000 - 1.050)*** | 0.022 |
| PM₂.₅ | SO₂ | 1.023 (0.976 - 1.071) | 0.094 | **1.032 (1.006 - 1.060)*** | 0.003 |
| PM₂.₅ | CO | 1.013 (0.961 - 1.067) | 0.067 | **1.028 (1.001 - 1.056)*** | 0.020 |
| NO₂ | **1.071 (1.029 - 1.114)** | | | | |
| NO₂ | PM₂.₅ | **1.085 (1.019 - 1.155)*** | 0.011 | **1.066 (1.016 - 1.119)*** | 0.010 |
| NO₂ | O₃ | 1.024 (0.972 - 1.078) | 0.376 | 1.037 (0.992 - 1.084) | 0.109 |
| NO₂ | SO₂ | **1.098 (1.022 - 1.180)*** | 0.011 | **1.067 (1.019 - 1.119)*** | 0.006 |
| NO₂ | CO | **1.096 (1.013 - 1.186)*** | 0.023 | **1.067 (1.016 - 1.120)*** | 0.010 |
| O₃ | **1.009 (1.001 - 1.016)** | | | | |
| O₃ | PM₂.₅ | 1.006 (0.997 - 1.015) | 0.157 | **1.008 (1.000 - 1.015)** | 0.059 |
| O₃ | NO₂ | 1.009 (0.998 - 1.019)* | 0.032 | 1.006 (0.998 - 1.014) | 0.074 |
| O₃ | SO₂ | 1.010 (0.998 - 1.021)* | 0.032 | **1.007 (1.000 - 1.015)** | 0.059 |
| O₃ | CO | 1.010 (0.997 - 1.023) | 0.076 | **1.008 (1.000 - 1.016)*** | 0.044 |
| SO₂ | 1.024 (0.982 - 1.067) | | | | |
| SO₂ | PM₂.₅ | 1.026 (0.970 - 1.085) | 0.613 | 1.036 (0.991 - 1.083) | 0.810 |
| SO₂ | NO₂ | 1.006 (0.942 - 1.074) | 0.938 | 1.015 (0.969 - 1.063) | 0.931 |
| SO₂ | O₃ | 1.027 (0.975 - 1.081) | 0.778 | 1.026 (0.982 - 1.072) | 0.888 |
| SO₂ | CO | 1.050 (0.984 - 1.121) | 0.612 | 1.035 (0.991 - 1.082) | 0.774 |
| CO | **1.007 (1.000 - 1.013)** | | | | |
| CO | PM₂.₅ | 1.000 (0.989 - 1.012) | 0.533 | 1.005 (0.995 - 1.015) | 0.796 |
| CO | NO₂ | 0.993 (0.979 - 1.008) | 0.135 | 1.005 (0.996 - 1.014) | 0.803 |
| CO | O₃ | **1.007 (1.000 - 1.014)** | 0.304 | **1.009 (1.001 - 1.016)** | 0.248 |
| CO | SO₂ | 0.999 (0.987 - 1.011) | 0.601 | 1.005 (0.997 - 1.013) | 0.594 |

^a^ Largest effect size chosen at lag 0 for PM_2.5_, lag 4 for NO₂ and CO, lag 1 for O₃, and lag 3 for SO₂.

^b^ Statistically significant results at p-value < 0.05 in bold.

* p-values for Cochran Q-test for heterogeneity

Figure S.6. Association between air pollutants and OHCA with a co-pollutant included as an interaction term (plot above) and as a confounder (plot below), by lag (days), adjusted for temperature and relative humidity. Results are expressed as Relative Risk (RR) and 95% Confidence Intervals (95% CI), per 10 µg/m³ increase in PM₂.₅, PM₁₀, NO₂, O₃; 100 µg/m³ increase in CO; and 1 µg/m³ increase in SO₂.


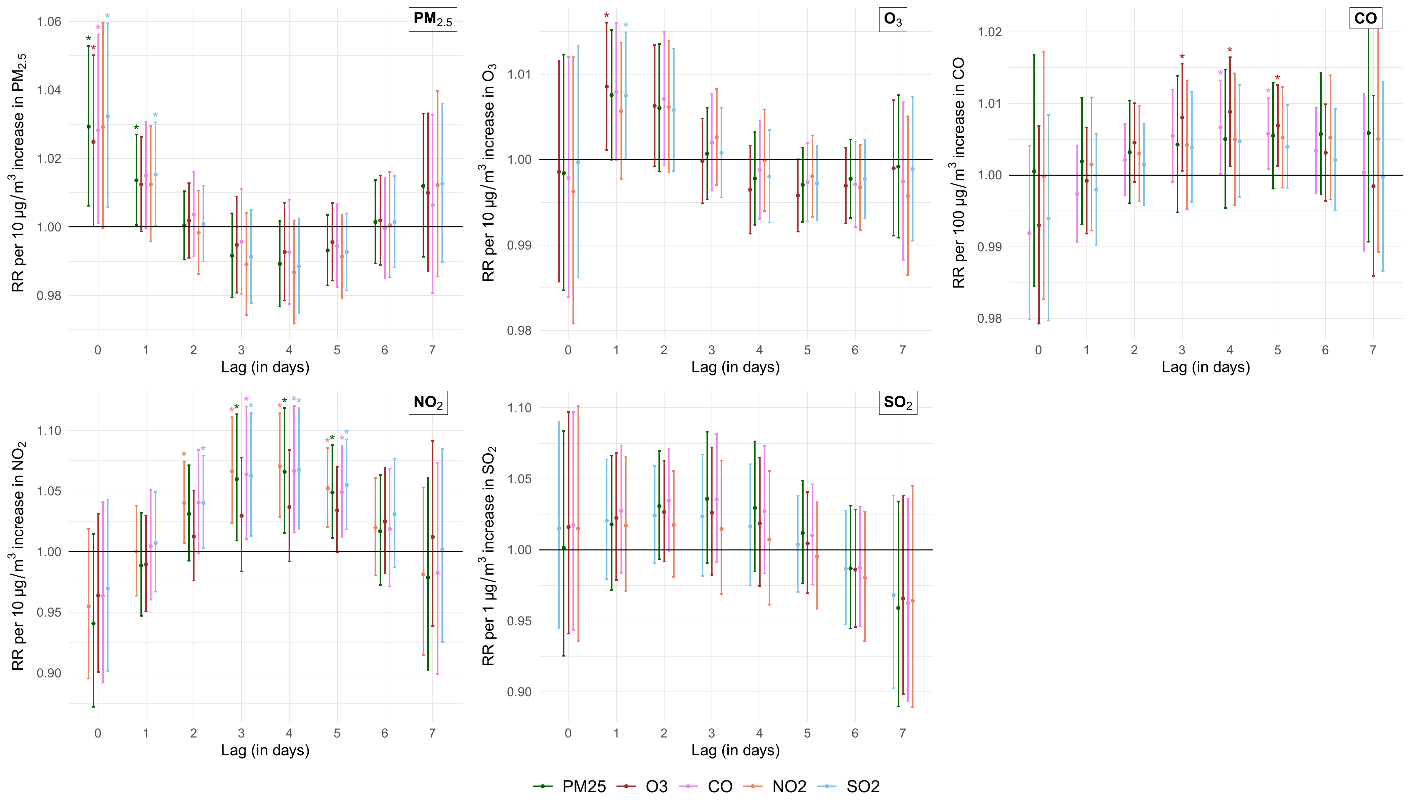


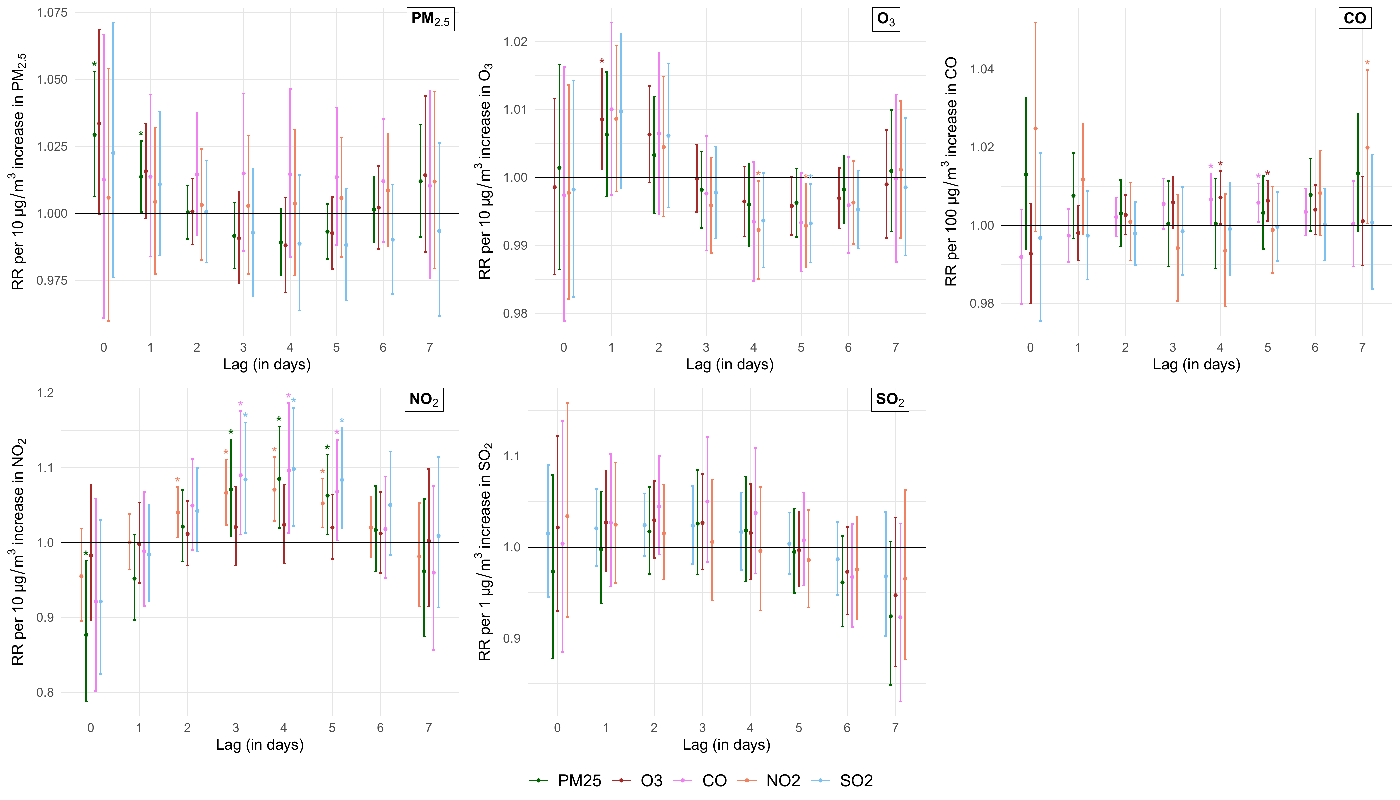


** Statistically significant results at p-value < 0.05 in bold.*

S.3. Literature review summary

Table S.6 : Case studies on association between air pollution and OHCA, referred to compare results of this study.

| Article | Years | Location | Design and Model | Spatial Confounders | Cases | RR (95%CI) | | | | | | Mean pollutants level | Units |
| --- | --- | --- | --- | --- | --- | --- | --- | --- | --- | --- | --- | --- | --- |
|  |  |  |  |  |  | PM₂.₅ | PM₁₀ | NO₂ | O₃ | SO₂ | CO |  |  |
| Gentile et al., 2021 ^1^ | 2019 | Pavia, Lodi, Cremona and Mantua (Milan, Italy) | Multivariable logistic regression model | Temperature and humidity | 1,922 | **2.6 (1.6–4)** | **2.7 (1.7-4.4)** | **2.8 (1.7-4.5)** | **2.4 (1.6-3.6)** | **4.1 (2.2-7.8)** | **3 (1.8-5)** | PM₂.₅ - 21.1 μg/m³ PM₁₀ - 29.6 μg/m³ NO₂ - 27.4 μg/m³ O₃ - 29.9 μg/m³ SO₂ - 3.2 μg/m³ CO - 0.5 mg/m³ | OR |
| Tobaldini et al., 2020 ^2^ | 2015 –2016 | Metropolitan Milan Area | Distributed lag non-linear model case-crossover analysis | Apparent temperature, relative humidity, influenza, PM₂.₅ | 5,761 |  | **1.7% (0.5%-2.9%) lag 3** |  |  |  |  | PM₂.₅ - 21.0 μg/m³ PM₁₀ - 29.0 μg/m³ | % change per 10 μg/m³ increase in pollutant |
| Moderato et al., 2023 ^3^ | 2010 -2017 | Piacenza, Italy | Time-stratified case-crossover conditional logistic regression | Temperature and seasons | 880 | 1.014 (0.021 - 1.007) | **1.011 (1.006 -1.016)** | **1.009 (1.004 -1.013)** | 0.997 (0.995 -1.000) |  | **1.037 (1.009 -1.066)** | PM₂.₅ - 23 μg/m³ PM₁₀ - 33 μg/m³ NO₂ - 42 μg/m³ O₃ - 43 μg/m³ CO - 5.32 μg/m³ | OR per 10 μg/m³ increase in pollutant |
| B. Zhao et al., 2020 ^4^ | 2014 -2015 | Japan | Time-stratified case-crossover design conditional logistic regression analysis | Temperature and humidity | 249,372 | **1.033 (1.023, 1.043) lag 0-3** |  | 0.987 (0.977, 0.998) lag1 | **1.011 (1.003, 1.019) lag 0-1** | **1.167 (1.075, 1.267) lag0-3** | **1.185 (1.095, 1.284) lag 0-3** | PM₂.₅ - 13.62 μg/m³ O₃ - 30.51 ppm NO₂ - 9.75 ppb SO₂ - 1.83 ppm CO - 0.35 ppm | OR per 10 μg/m³ increase in pollutant |
| Dennekamp et al., 2010 ^5^ | 2003 - 2006 | Melbourne, Australia | Time-stratified case-crossover conditional logistic regression | Temperature and humidity | 8434 | **3.61 (1.29 - 5.99) lag 01** | **-2.91 ( - 5.15 - - 0.62) lag 3** | 2.99 ( -2.57 - 8.86) lag 01 | 3.78 (-1.32 - 9.14) lag 0 | 3.24 ( - 0.59 - 7.22) lag 3 | **3.09 (0.25 - 6.02) lag 01** | PM₂.₅ - 6.35 μg/m³ PM₁₀ - 19.65 μg/m³ NO₂ - 12.00 ppb O₃ - 13.34 ppb SO₂ - 0.49 ppb CO - 0.44 ppm | % change per IQR increase in pollutant |
| Dai et al., 2015 ^6^ | 2011–2013 | Shenzhen, China | Over-dispersed generalized linear Poisson models | Temperature, relative humidity and co-pollutants | 1975 |  | 6.31 (−2.83 - 15.45) lag 1 | **8.01 (1.07 - 14.95) lag 1** | 7.49 (−2.18 - 17.16) lag 0 | **7.24 (0.76 - 13.73) lag 2** | 3.50 (−7.36 - 14.36) lag 1 | PM₁₀ - 57.85 μg/m³ NO₂ - 47.53 μg/m³ O₃ - 55.75 μg/m³ SO₂ - 10.79 μg/m³ CO - 1020 μg/m³ | % change associated with an IQR increase in pollutant |
| Yorifuji et al., 2014 ^7^ | 2006 - 2010 | Okayama, Japan | Time-stratified case-crossover design conditional logistic regression | Ambient temperature, relative humidity | 558 |  | **1.17 (1.02–1.33) lag 2** | **1.24 (1.01–1.53) lag 1** | **1.40 (1.02–1.92) lag 3** | **1.16 (1.00–1.34) lag 2** | 1.15 (0.96–1.37) lag 1 | PM₁₀ - 26.8 μg/m³ NO₂ - 17.1 ppb O₃ - 25.9 ppb SO₂ - 3.0 ppb CO - 0.6 ppm | Adjusted Odds Ratio per IQR increase in pollutant |
| Raza et al., 2014 ^8^ | 2000 -2010 | Stockholm County, Sweden | Time-stratified case-crossover design conditional logistic regression | Temperature and relative humidity | 5973 | 1.01 (0.95-1.07) lag 0 | 0.99 (0.93-1.05) lag 0 | 0.96 (0.89 - 0.99) lag 0 | **1.05 (1.01–1.09) lag 3** |  |  | PM₂.₅ - 8.1 μg/m³ PM₁₀ -7.2 μg/m³ NO₂ - 15.7 μg/m³ O₃ urban - 51.2 μg/m³ O₃ rural - 60.0 μg/m³ | Odds Ratio per 10 μg/m³ increase in pollutant |
| Dahlquist et al., 2023 ^9^ | 2009 -2019 | Sweden | Time-stratified case-crossover | Temperature and co-pollutants | 29,604 | **1.070 (1.018, 1.125) lag 4** | **1.038 (1.009, 1.069) lag 4** | 1.009 (0.975, 1.044) lag 6 | 1.023 (0.995, 1.051) lag 0 |  |  | PM₂.₅ - 7.2 μg/m³ PM₁₀ - 13.7 μg/m³ O₃ - 54.2 μg/m³ NO₂ - 11.4 μg/m³ | % change in RR per 10 μg/m³ increase in pollutant |
| Kim et al., 2020 ^10^ | 2012 - 2016 | South Korea | Multivariable generalised additive model | Average temperature, daily temperature range, humidity | 38,928 | **1.0159 (1.0151 - 1.0166) lag 1** |  |  |  |  |  | PM₂.₅ - 15.6 μg/m³ | RR per 10 μg/m³ increase in pollutant |
| Ensor et al., 2013 ^11^ | 2004 -2011 | Houston, Texas | Time-stratified case-crossover conditional logistic regression | Apparent temperature | 11,677 | **4.6 (1.2 - 8.2) lag 1-2** |  | 0.9 (-2.8 - 4.7) lag 3 | **3.8 (0.4 - 7.2) lag 0** | -1.3 (-3.7 - 1.1) lag 1-2 | 1.8 (-0.9 - 4.6) lag 0 | PM₂.₅ - 11.42 μg/m³ NO₂ - 9.11 ppb O₃ - 25.52 ppb SO₂ - 1.96 ppb CO - 280 ppb | % change associated with an IQR increase in pollutant |
| Silverman et al., 2010 ^12^ | 2002 -2006 | New York | Time-stratified case-crossover Poisson generalized linear model | Season, apparent temperature | 8,216 | **1.06 (1.01 - 1.1)** |  | 1.02(0.97-1.06) | 1.04(0.98-1.10) | 1.00(0.96-1.05) | 0.99(0.95-1.03) | PM₂.₅ - 12 μg/m³ NO₂ - 27 ppb O₃ - 28 ppb SO₂ - 6.3 ppb CO - 0.9 ppm | RR per IQR increase in pollutant |
| Cheng et al., 2020 ^13^ | 2015 -2016 | Kaohsiung, Taiwan | Time-stratified case-crossover conditional logistic regression | Temperature and humidity | 3,566 | **1.11 (1.01-1.2) lag 1** | **1.11 (1.01 -1.22) lag 2** | **1.05(1.001-1.03) lag 3** |  | 1.005(0.98-1.02) lag 1 | **1.06 (1.003-1.12) lag 1** | PM₂.₅ - 25.2 μg/m³ PM₁₀ - 58.3 μg/m³ NO₂ - 16.5 ppb O₃ - 27.4 ppb SO₂ - 4.4 ppb | OR associated with an IQR increase in pollutant |
| Wichmann et al., 2013 ^14^ | 2000 –2010 | Copenhagen, Denmark | Time-stratified case-crossover Poisson Generalized Additive Model | Temperature, relative humidity and co-pollutants | 4,657 | **5.2% (1.0% - 9.5%) lag 4** | **4.7% (0.7% - 8.8%) lag 3** | NOx: 3.4% (0.9% - 7.9%) lag 3 |  |  |  | PM₂.₅ - 10.31 μg/m³ PM₁₀ - 15.73 μg/m³ NOx - 14.75 ppb O₃ - 25.94 ppb | % change per IQR increase in pollutant |
| Rosenthal et al., 2013 ^15^ | 1998 –2006 | Helsinki, Finland | Case-crossover conditional logistic regression | Temperature and humidity | 2,134 | **1.07 (1.01–1.13) lag 0-1 hr** | **1.05 (1.00 –1.09) lag 1 hr** | 0.89 (0.75–1.06) lag 03 | **1.18 (1.03 –1.35) lag 2** | 0.95 (0.89–1.01) lag 1 | 1.07 (0.94–1.21) lag 03 | PM₂.₅ - 8.7 μg/m³ PM₁₀ - 19.4 μg/m³ NO₂ - 24.1 μg/m³ O₃ - 46.8 μg/m³  SO₂ - 3.8 μg/m³ CO - 0.35 mg/m³ | OR per IQR increase in pollutant |
| Straney et al., 2014 ^16^ | 2000 -2010 | Perth, Australia | Case-crossover conditional logistic regression | Mean hourly temperature and relative humidity | 8,551 | **1.010 (1.001 - 1.018) lag 0-2** | 1.002 (0.999 - 1.006) lag 0-1 | 1.002 (0.998 - 1.007) lag 0 | 1.002 (0.997 - 1.007) lag 0-1 | 1.001 (0.991 - 1.010) lag 1 | 1.114 (1.010 - 1.228) lag 12 hrs | PM₂.₅ - 6.80 μg/m³ PM₁₀ - 14.60 μg/m³ NO₂ - 3.00 ppb O₃ - 20.00 ppb SO₂ - 0.40 ppb CO - 0.11 ppb | OR per unit increase in pollutant concentration |
| Wang Y C et al., 2021 ^17^ | 2006 - 2015 | Taiwan | Distributed lag non-linear model time-series analysis | Temperature, wind speed and humidity | 117,488 | 0.03% (95% CI; -0.013, 0.07) |  |  | **0.07% (95% CI; 0.01, 0.14)** |  |  | PM₂.₅ - 29.6 μg/m³  O₃ - 29.4 ppb | % change per at 90 percentiles |
| Kang et al, 2016 ^18^ | 2006 - 2013 | Seoul, South Korea | Case-crossover and time-series conditional logistic regression | Temperature, relative  humidity and air pressure | 28,315 | **1.13%; (95% CI, 0.16–2.11) lag 2** | **0.48 % (95% CI, 0.06 – 0.89) lag 2** | **1.31% (95% CI, 0.08 - 2.54) lag 1** | 0.96% (95% CI, -0.25 - 2.18) lag 2 | **0.98% (95% CI, 0.23 - 1.73) lag 1** | **0.91 % (95% CI, 0.23 - 1.59) lag 1** | PM₂.₅ - 25.8 μg/m³  PM₁₀ - 52.7 μg/m³  NO₂ - 37.9 ppb  O₃ - 29.9 ppb  SO₂ - 5.6 ppb  CO - 0.61 ppm | ER per 10 units increase in PM, NO₂, and O₃, 1 ppb in SO₂ and 0.1 ppm in CO |
| Our Study | 2016 - 2019 | Lombardy, Italy | Distributed lag non-linear model case-crossover analysis | Temperature and humidity | 37,613 | **1.029 (1.006 - 1.053 ) lag 0** | **1.025 (1.005 - 1.046) lag 0** | **1.071 (1.029 - 1.114)**  **lag 4** | **1.009 (1.001 - 1.016)**  **lag 1** | 1.024 (0.990 - 1.059) lag 2 | **1.007 (1.0002 - 1.013 ) lag 4** | PM₂.₅ - 21.42 μg/m³ PM₁₀ - 25.88 μg/m³ NO₂ - 26.05 μg/m³ O₃ - 49.28 μg/m³ SO₂ - 2.25 μg/m³ CO - 302.5 μg/m³ | RR per 10 µg/m³ increase (SO₂ per 1 µg/m³, CO per 100 µg/m³) |

Table S.7 : Meta-analysis on association between air pollution and OHCA, referred to compare results of this study.

| Meta-analysis | RR (95%CI) | | | | | | Units |
| --- | --- | --- | --- | --- | --- | --- | --- |
|  | PM₂.₅ | PM₁₀ | NO₂ | O₃ | SO₂ | CO |  |
| R. Zhao et al., 2017 ^19^ | **1.04 (1.01 - 1.07)** | **1.02 (1.01 - 1.04)** | **1.02 (1.00 - 1.03)** | **1.02 (1.01 - 1.02)** | 1.01 (0.99 - 1.03) | 1.06 (1.00 - 1.14) | RR per 10 μg/m³ increase in pollutant |
| Sangkharat et al., 2019 ^20^ | Ambulance dispatch data: 1.02 (0.99 - 1.05) |  |  |  |  |  | RR per 10 μg/m³ increase in pollutant for PM, 1 ppm of CO, 10 ppb of SO₂, NO₂ and O₃ |
|  | **Paramedic assessment data: 1.05 (1.03 - 1.08)** | 1.02 (1.00 - 1.05) | 1.00 (0.96-1.04) | 1.02 (1.00 - 1.05) | 1.03 (0.94 - 1.12) | **1.10 (1.02 - 1.18)** |  |
| Our Study | **1.029 (1.006 - 1.053 ) lag 0** | **1.025 (1.005 - 1.046) lag 0** | **1.071 (1.029 - 1.114)**  **lag 4** | **1.009 (1.001 - 1.016)**  **lag 1** | 1.024 (0.990 - 1.059) lag 2 | **1.007 (1.0002 - 1.013) lag 4** | RR per 10 µg/m³ increase (SO₂ per 1 µg/m³, CO per 100 µg/m³) |

Note - Statistically significant results at *p-value* < 0.05 in bold.

# S.4. Sensitivity analysis

Figure S.7: Sensitivity analyses for individual relative risks linked to OHCA per 10 µg/m³ increase in PM₂.₅, PM₁₀, NO₂, O₃; per 100 µg/m³ increase in CO; and per 1 µg/m³ increase in SO₂ at lag 0-3, lag 0-7, lag 0-10 and lag 0-14 days.


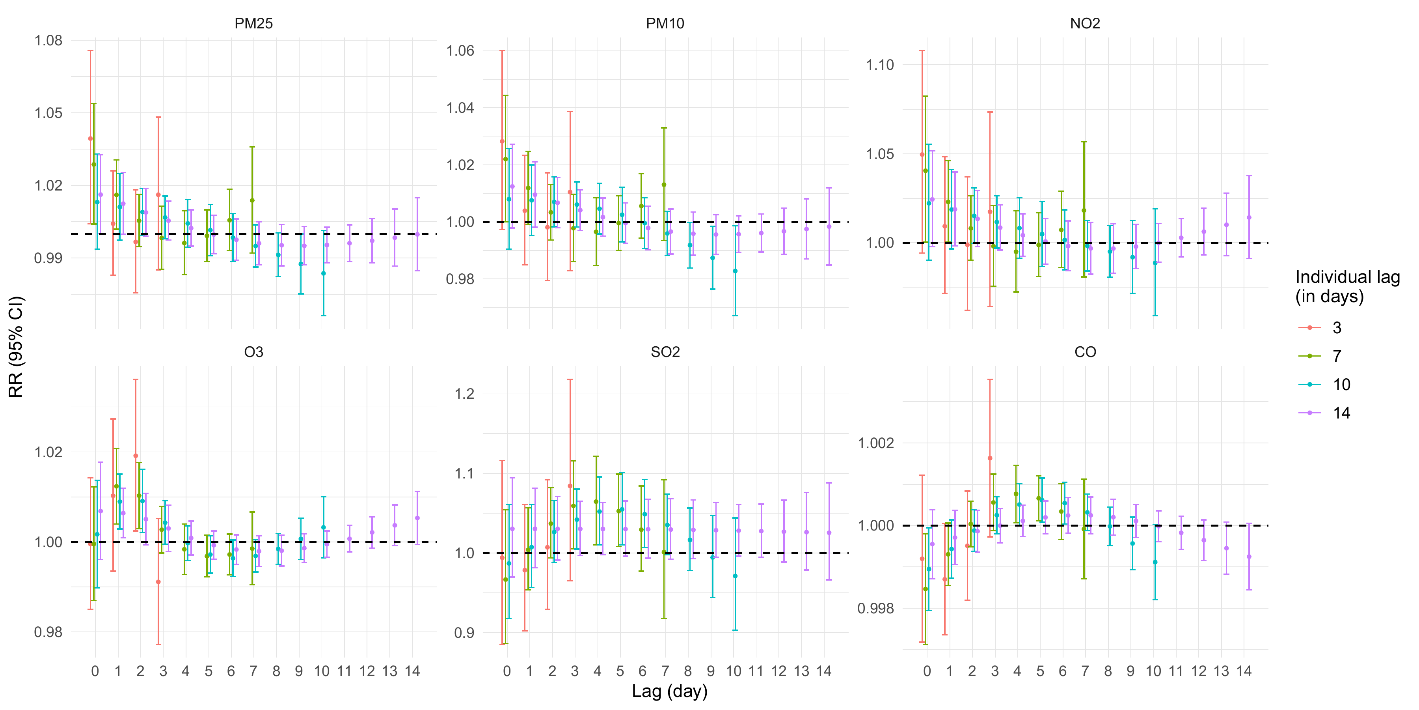
Figure S.8: Sensitivity analyses for cumulative relative risks linked to OHCA per 10 µg/m³ increase in PM₂.₅, PM₁₀, NO₂, O₃; per 100 µg/m³ increase in CO; and per 1 µg/m³ increase in SO₂ at lag 0-3, lag 0-7, lag 0-10 and lag 0-14 days.


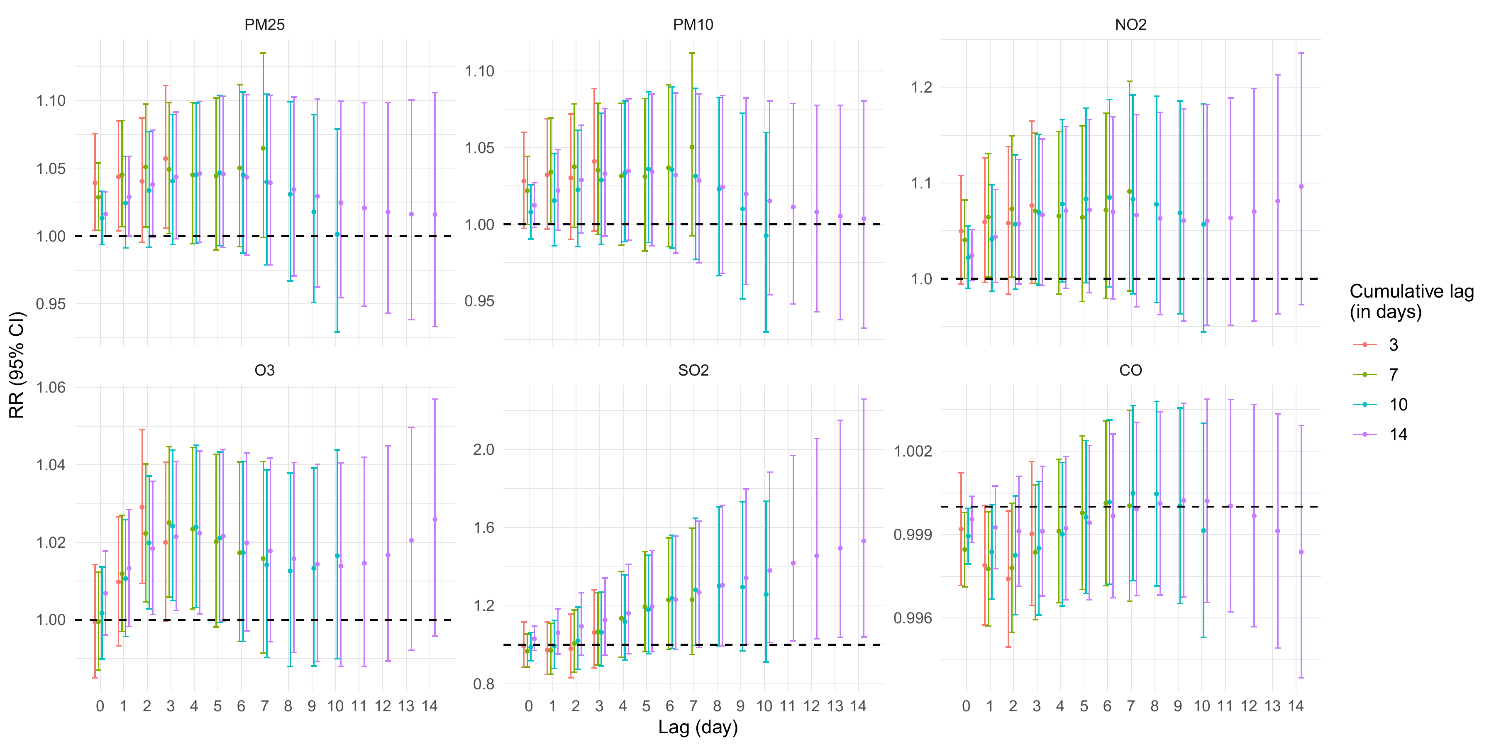


Table S.8: Comparative performance of models with varying lag length of the pollutants.

| Pollutant | Lag Length  (in days) | QAIC ^a^ | QAIC_max - QAIC (in %) | RR (95% CI) ^b^  (individual lag) | RR (95% CI) ^b^  (cumulative lag) |
| --- | --- | --- | --- | --- | --- |
| PM₂.₅ | 3 | 8517 | -0.139 | 1.016 (0.985-1.048) | 1.057 (1.006-1.111) |
| **PM₂.₅** | **7** | **8515** | **-0.159** | **1.014 (0.992-1.036)** | **1.065 (0.999-1.135)** |
| PM₂.₅ | 10 | 8517 | -0.13 | 0.984 (0.966-1.001) | 1.001 (0.929-1.079) |
| PM₂.₅ | 14 | 8529 | 0 | 1.000 (0.985-1.015) | 1.016 (0.933-1.106) |
| PM₁₀ | 3 | 8523 | -0.091 | 1.010 (0.983-1.039) | 1.041 (0.996-1.089) |
| **PM₁₀** | **7** | **8520** | **-0.12** | **1.013 (0.994-1.033)** | **1.050 (0.993-1.112)** |
| PM₁₀ | 10 | 8520 | -0.116 | 0.983 (0.967-0.999) | 0.993 (0.930-1.060) |
| PM₁₀ | 14 | 8530 | 0 | 0.998 (0.985-1.012) | 1.004 (0.932-1.081) |
| NO₂ | 3 | 8505 | -0.201 | 1.071 (0.946-1.212) | 0.973 (0.814-1.162) |
| **NO₂** | **7** | **8520** | **-0.025** | **1.013 (0.933-1.100)** | **1.002 (0.787-1.275)** |
| NO₂ | 10 | 8514 | -0.097 | 1.032 (0.959-1.110) | 1.077 (0.811-1.431) |
| NO₂ | 14 | 8522 | 0 | 1.025 (0.972-1.081) | 1.124 (0.830-1.524) |
| O₃ | 3 | 8516 | -0.184 | 0.980 (0.957-1.003) | 1.016 (0.985-1.049) |
| **O₃** | **7** | **8529** | **-0.024** | **0.996 (0.981-1.010)** | **1.012 (0.974-1.051)** |
| O₃ | 10 | 8529 | -0.024 | 1.007 (0.994-1.020) | 1.021 (0.980-1.064) |
| O₃ | 14 | 8531 | 0 | 1.007 (0.997-1.017) | 1.026 (0.978-1.076) |
| SO₂ | 3 | 8533 | 0 | 1.084 (0.965-1.218) | 1.062 (0.881-1.280) |
| **SO₂** | **7** | **8520** | **-0.146** | **1.001 (0.918-1.092)** | **1.231 (0.949-1.598)** |
| SO₂ | 10 | 8522 | -0.124 | 0.971 (0.903-1.044) | 1.257 (0.912-1.735) |
| SO₂ | 14 | 8526 | -0.081 | 1.025 (0.966-1.088) | 1.532 (1.039-2.259) |
| CO | 3 | 8520 | -0.011 | 1.003 (1.000-1.005) | 0.999 (0.996-1.002) |
| **CO** | **7** | **8519** | **-0.021** | **1.000 (0.999-1.001)** | **1.000 (0.997-1.003)** |
| CO | 10 | 8521 | 0 | 0.999 (0.998-1.000) | 0.999 (0.995-1.003) |
| CO | 14 | 8521 | -0.003 | 0.999 (0.999-1.000) | 0.998 (0.993-1.002) |

^a^ Quasi-Akaike’s information criterion for quasi-Poisson (Q-AIC).

^b^ Individual and consecutive relative risks (RR) at the final lag day of the respective lag length.

Figure S.9: Sensitivity analyses for relative risks up from lag 0 to lag 7 linked to OHCA per 10 µg/m³ increase in PM₂.₅, PM₁₀, NO₂, O₃; per 100 µg/m³ increase in CO; and per 1 µg/m³ increase in SO₂ by changing the smoothing functions and degree of freedom of predictor (pollutant) and its lag structure in the crossbasis included in the models.


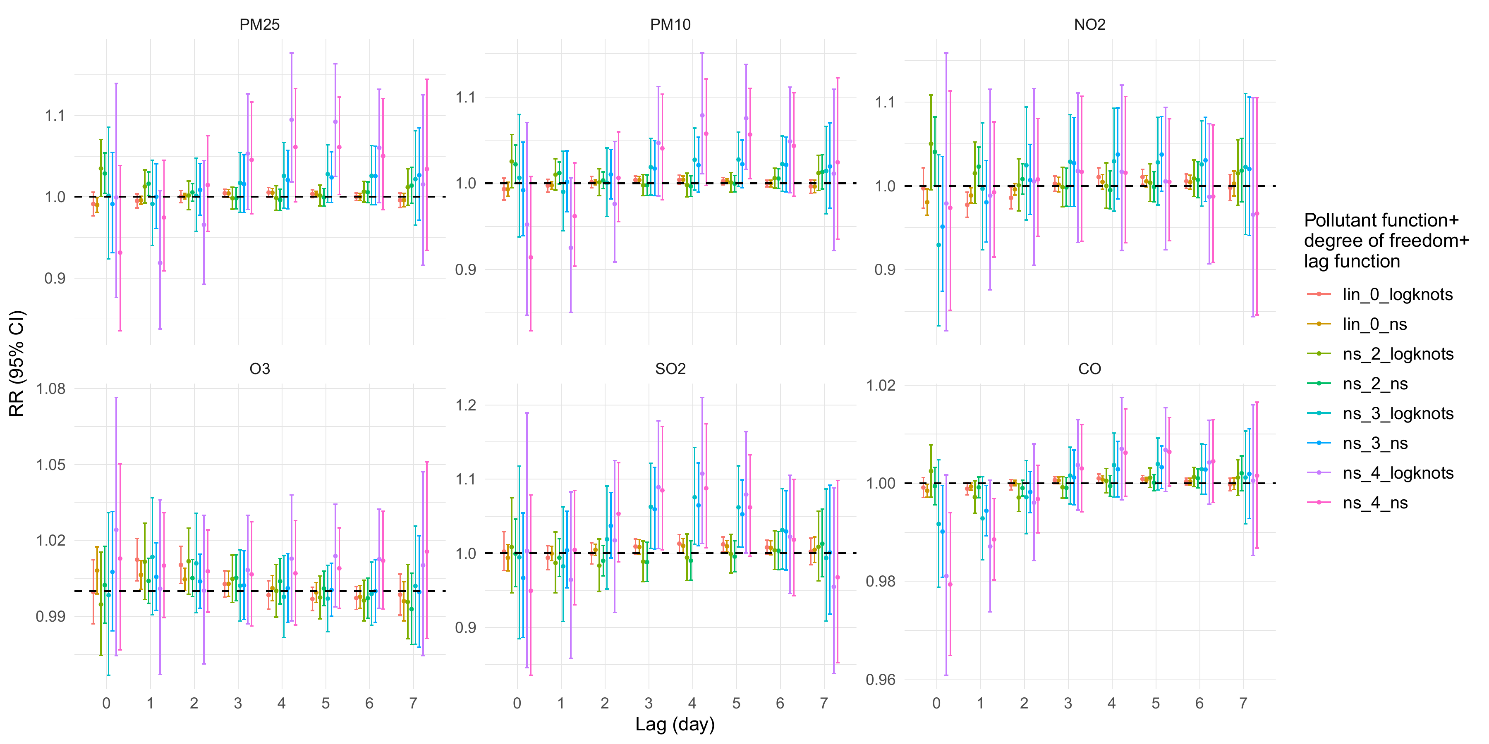


Table S.9: Comparative performance of models with combinations of smoothing functions and lag structures of air pollutants.

| Pollutant | Parameter combination ^a^ | QAIC ^b^ | QAIC_max - QAIC (in %) | RR (95% CI) ^c^ |  | Pollutant | QAIC ^b^ | QAIC_max - QAIC (in %) | RR (95% CI) ^c^ |
| --- | --- | --- | --- | --- | --- | --- | --- | --- | --- |
| PM₂.₅ | lin-0-logknots | 8524 | 0.000 | 0.991 (0.977-1.006) |  | O₃ | 8521 | -0.201 | 1.010 (1.003-1.018) |
| PM₂.₅ | lin-0-ns | 8522 | -0.024 | 0.990 (0.981-0.999) |  | O₃ | 8521 | -0.198 | 1.006 (1.001-1.012) |
| PM₂.₅ | ns-2-logknots | 8519 | -0.055 | 1.035 (1.000-1.070) |  | O₃ | 8529 | -0.102 | 1.010 (0.995-1.024) |
| PM₂.₅ | ns-2-ns | 8515 | -0.103 | 1.029 (1.004-1.054) |  | O₃ | 8527 | -0.126 | 1.006 (0.996-1.015) |
| PM₂.₅ | ns-3-logknots | 8523 | -0.006 | 1.001 (0.924-1.085) |  | O₃ | 8537 | -0.009 | 1.011 (0.989-1.034) |
| PM₂.₅ | ns-3-ns | 8517 | -0.077 | 0.991 (0.931-1.055) |  | O₃ | 8533 | -0.060 | 1.008 (0.994-1.022) |
| PM₂.₅ | ns-4-logknots | 8524 | -0.005 | 0.999 (0.876-1.139) |  | O₃ | 8538 | 0.000 | 1.001 (0.967-1.036) |
| PM₂.₅ | ns-4-ns | 8520 | -0.047 | 0.931 (0.836-1.038) |  | O₃ | 8535 | -0.030 | 1.013 (0.992-1.034) |
| PM₁₀ | lin-0-logknots | 8526 | -0.039 | 0.993 (0.981-1.006) |  | SO₂ | 8523 | -0.115 | 1.009 (0.999-1.019) |
| PM₁₀ | lin-0-ns | 8524 | -0.063 | 0.993 (0.985-1.001) |  | SO₂ | 8522 | -0.129 | 1.008 (0.998-1.018) |
| PM₁₀ | ns-2-logknots | 8524 | -0.055 | 1.025 (0.995-1.057) |  | SO₂ | 8528 | -0.054 | 0.988 (0.961-1.016) |
| PM₁₀ | ns-2-ns | 8520 | -0.104 | 1.022 (1.000-1.044) |  | SO₂ | 8525 | -0.094 | 0.987 (0.961-1.014) |
| PM₁₀ | ns-3-logknots | 8528 | -0.011 | 1.006 (0.938-1.080) |  | SO₂ | 8526 | -0.084 | 1.063 (1.007-1.121) |
| PM₁₀ | ns-3-ns | 8522 | -0.079 | 0.992 (0.939-1.048) |  | SO₂ | 8520 | -0.148 | 1.059 (1.006-1.116) |
| PM₁₀ | ns-4-logknots | 8529 | 0.000 | 0.952 (0.847-1.070) |  | SO₂ | 8533 | 0.000 | 1.089 (1.006-1.179) |
| PM₁₀ | ns-4-ns | 8522 | -0.077 | 0.914 (0.829-1.008) |  | SO₂ | 8526 | -0.086 | 1.085 (1.005-1.171) |
| NO₂ | lin-0-logknots | 8518 | -0.075 | 1.011 (1.000-1.022) |  | CO | 8521 | -0.133 | 1.001 (1.000-1.002) |
| NO₂ | lin-0-ns | 8520 | -0.053 | 1.005 (0.996-1.013) |  | CO | 8519 | -0.150 | 1.001 (1.000-1.001) |
| NO₂ | ns-2-logknots | 8520 | -0.059 | 0.997 (0.970-1.024) |  | CO | 8525 | -0.089 | 1.000 (0.998-1.003) |
| NO₂ | ns-2-ns | 8519 | -0.069 | 0.992 (0.969-1.015) |  | CO | 8523 | -0.106 | 0.999 (0.997-1.002) |
| NO₂ | ns-3-logknots | 8520 | -0.060 | 1.022 (0.963-1.084) |  | CO | 8527 | -0.059 | 1.004 (0.997-1.010) |
| NO₂ | ns-3-ns | 8518 | -0.084 | 1.028 (0.975-1.083) |  | CO | 8524 | -0.095 | 1.003 (0.997-1.009) |
| NO₂ | ns-4-logknots | 8525 | 0.000 | 1.016 (0.923-1.119) |  | CO | 8532 | 0.000 | 1.007 (0.997-1.017) |
| NO₂ | ns-4-ns | 8521 | -0.047 | 1.013 (0.930-1.102) |  | CO | 8527 | -0.065 | 1.006 (0.997-1.015) |

^a^ Evaluated transformation functions of air pollutants: natural spine with 2 to 4 degrees of freedom and linear. Lag structures evaluated with natural spline with 2 degrees of freedom and ‘logknots’ function, with 2 knots placed at equally spaced quantiles.

^b^ Quasi-Akaike’s information criterion for quasi-Poisson (Q-AIC).

^c^ Relative risk (95% ci) is reported for lag with the largest RR per 10 µg/m³ increase in PM₂.₅, NO₂, O₃; per 100 µg/m³ increase in CO; and per 1 µg/m³ increase in SO₂ up to lag day 7. Largest effect size chosen at lag 0 for PM₂.₅ and PM₁₀, lag 4 for NO₂ and CO, lag 1 for O₃, and lag 3 for SO₂.

Figure S.10: Sensitivity analyses for relative risks up from lag 0 to lag 7 linked to OHCA per 10 µg/m³ increase in PM₂.₅, PM₁₀, NO₂, O₃; per 100 µg/m³ increase in CO; and per 1 µg/m³ increase in SO₂ by changing the knot placement on the natural spline function of the predictor (temperature) and its lag structure up to lag 14 in the crossbasis included in the models.


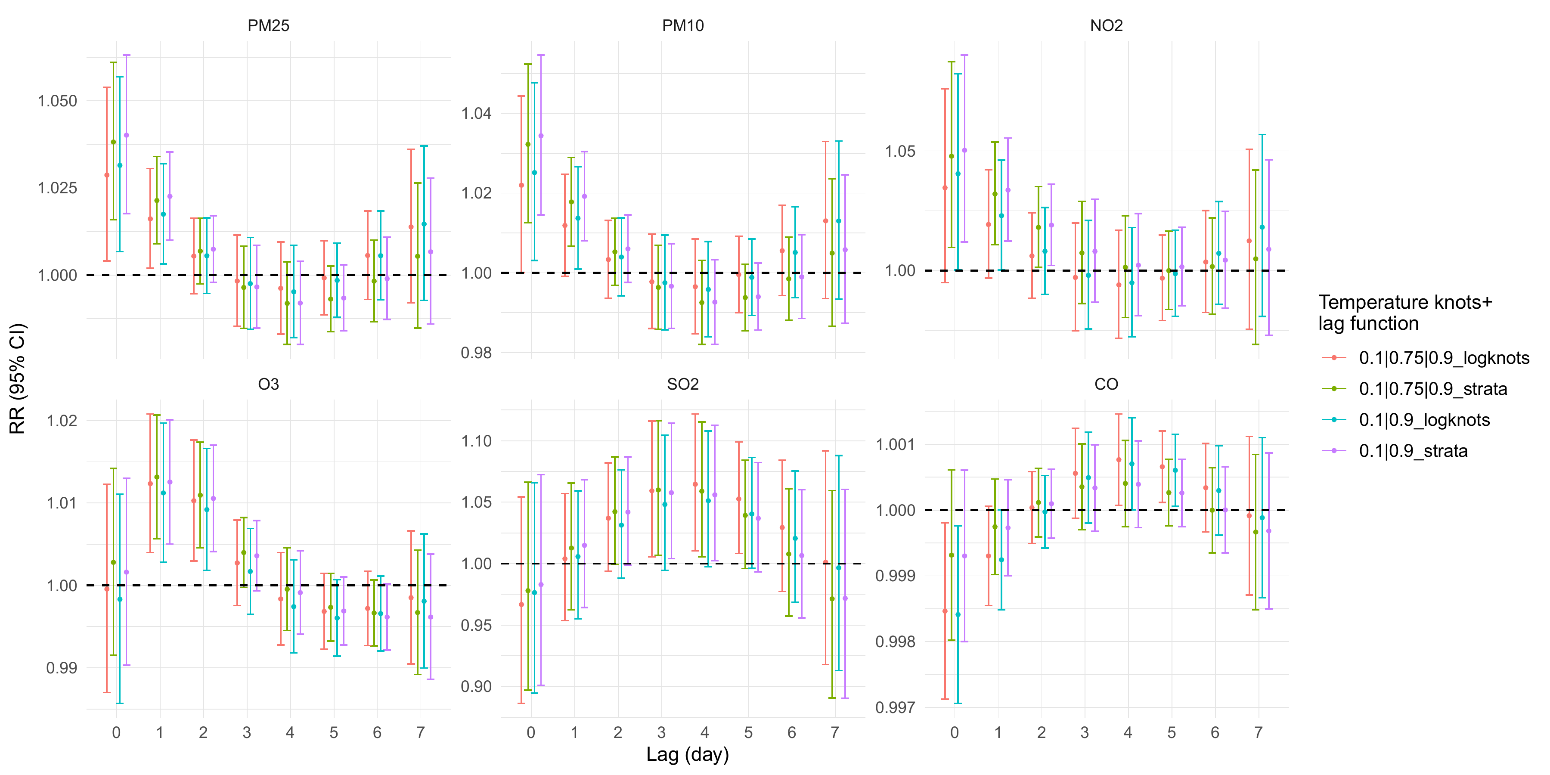


Figure S.11: Sensitivity analyses for relative risks up from lag 0 to lag 7 linked to OHCA per 10 µg/m³ increase in PM₂.₅, PM₁₀, NO₂, O₃; per 100 µg/m³ increase in CO; and per 1 µg/m³ increase in SO₂ by changing the length of the running mean of the relative humidity and the degree of freedom of the natural spline in its onebasis included in the models.


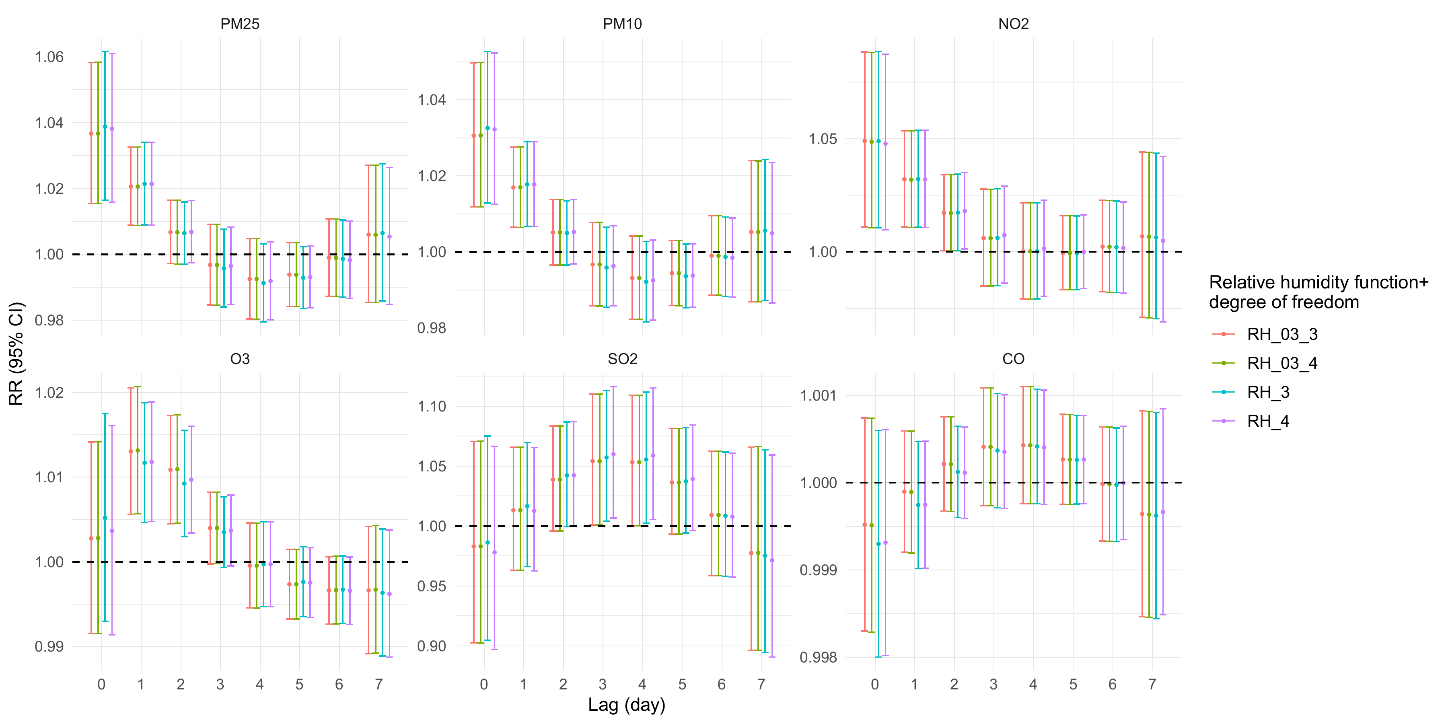


Table S.10: Comparative performance of models with varying temporal adjustment structures.

| Model | Pollutant | RR^b^ | 95% CI | p-value | QAIC^c^ |
| --- | --- | --- | --- | --- | --- |
| Adjusted for time trend^a^, DOW, holidays | PM₂.₅ | 1.026 | 1.001 - 1.052 | 0.040 | 8212 |
| Adjusted for DOW, holidays |  | 1.027 | 1.002 - 1.052 | 0.036 | 8209 |
| Adjusted for time trend, holidays |  | 1.026 | 1.001 - 1.052 | 0.040 | 8200 |
| Adjusted for time trend, DOW |  | 1.027 | 1.002 - 1.053 | 0.034 | 8215 |
| Adjusted for time trend |  | 1.027 | 1.002 - 1.053 | 0.034 | 8203 |
| **Adjusted for holidays** |  | **1.027** | **1.002 - 1.052** | **0.036** | **8197** |
| Unadjusted |  | 1.028 | 1.003 - 1.053 | 0.030 | 8200 |
| Adjusted for time trend, DOW, holidays | PM₁₀ | 1.019 | 0.997 - 1.042 | 0.087 | 8217 |
| Adjusted for DOW, holidays |  | 1.020 | 0.998 - 1.043 | 0.075 | 8214 |
| Adjusted for time trend, holidays |  | 1.019 | 0.997 - 1.042 | 0.087 | 8205 |
| Adjusted for time trend, DOW |  | 1.020 | 0.998 - 1.043 | 0.076 | 8220 |
| Adjusted for time trend |  | 1.020 | 0.998 - 1.043 | 0.076 | 8208 |
| **Adjusted for holidays** |  | **1.020** | **0.998 - 1.043** | **0.075** | **8202** |
| Unadjusted |  | 1.021 | 0.999 - 1.044 | 0.065 | 8204 |
| Adjusted for time trend, DOW, holidays | NO₂ | 1.028 | 0.976 - 1.083 | 0.298 | 8216 |
| Adjusted for DOW, holidays |  | 1.028 | 0.976 - 1.083 | 0.300 | 8212 |
| Adjusted for time trend, holidays |  | 1.028 | 0.976 - 1.083 | 0.298 | 8204 |
| Adjusted for time trend, DOW |  | 1.031 | 0.979 - 1.087 | 0.244 | 8218 |
| Adjusted for time trend |  | 1.031 | 0.979 - 1.087 | 0.244 | 8206 |
| **Adjusted for holidays** |  | **1.028** | **0.976 - 1.083** | **0.300** | **8200** |
| Unadjusted |  | 1.031 | 0.979 - 1.087 | 0.244 | 8202 |
| Adjusted for time trend, DOW, holidays | O₃ | 1.010 | 1.003 - 1.018 | 0.008 | 8219 |
| Adjusted for DOW, holidays |  | 1.010 | 1.002 - 1.018 | 0.010 | 8215 |
| Adjusted for time trend, holidays |  | 1.010 | 1.003 - 1.018 | 0.008 | 8206 |
| Adjusted for time trend, DOW |  | 1.011 | 1.003 - 1.019 | 0.007 | 8221 |
| Adjusted for time trend |  | 1.011 | 1.003 - 1.019 | 0.007 | 8209 |
| **Adjusted for holidays** |  | **1.010** | **1.002 - 1.018** | **0.010** | **8203** |
| Unadjusted |  | 1.010 | 1.003 - 1.018 | 0.009 | 8205 |
| Adjusted for time trend, DOW, holidays | SO₂ | 1.053 | 0.999 - 1.109 | 0.055 | 8223 |
| Adjusted for DOW, holidays |  | 1.051 | 0.998 - 1.107 | 0.062 | 8218 |
| Adjusted for time trend, holidays |  | 1.053 | 0.999 - 1.109 | 0.055 | 8211 |
| Adjusted for time trend, DOW |  | 1.051 | 0.997 - 1.108 | 0.064 | 8226 |
| Adjusted for time trend |  | 1.051 | 0.997 - 1.108 | 0.064 | 8213 |
| **Adjusted for holidays** |  | **1.051** | **0.998 - 1.107** | **0.062** | **8206** |
| Unadjusted |  | 1.049 | 0.996 - 1.106 | 0.070 | 8209 |
| Adjusted for time trend, DOW, holidays | CO | 1.001 | 1.000 - 1.002 | 0.003 | 8214 |
| Adjusted for DOW, holidays |  | 1.001 | 1.000 - 1.002 | 0.003 | 8211 |
| Adjusted for time trend, holidays |  | 1.001 | 1.000 - 1.002 | 0.003 | 8202 |
| Adjusted for time trend, DOW |  | 1.001 | 1.000 - 1.002 | 0.004 | 8218 |
| Adjusted for time trend |  | 1.001 | 1.000 - 1.002 | 0.004 | 8206 |
| **Adjusted for holidays** |  | **1.001** | **1.000 - 1.002** | **0.003** | **8199** |
| Unadjusted |  | 1.001 | 1.000 - 1.002 | 0.004 | 8202 |

^a^ Based on the preliminary sensitivity analysis, long-term time trend was tested using linear and natural splines with 2 and 3 degrees of freedom. Date of week (DOW) are added as categorial values, and holidays as binary.

^b^ Relative risk (95% ci) is reported for lag with the largest RR per 10 µg/m³ increase in PM₂.₅, NO₂, O₃; per 100 µg/m³ increase in CO; and per 1 µg/m³ increase in SO₂ up to lag day 7. Largest effect size chosen at lag 0 for PM₂.₅ and PM₁₀, lag 4 for NO₂ and CO, lag 1 for O₃, and lag 3 for SO₂.

^c^ Quasi-Akaike’s information criterion for quasi-Poisson (Q-AIC).

Note – selected model in bold.

# References

1. Gentile FR, Primi R, Baldi E, Compagnoni S, Mare C, Contri E, et al. Out-of-hospital cardiac arrest and ambient air pollution: A dose-effect relationship and an association with OHCA incidence. PLoS One. 2021 Jun;16(8):e0256526.

2. Tobaldini E, Iodice S, Bonora R, Bonzini M, Brambilla A, Sesana G, et al. Out-of-hospital cardiac arrests in a large metropolitan area: synergistic effect of exposure to air particulates and high temperature. Eur J Prev Cardiol. 2020 Jun;27(5):513–9.

3. Moderato L, Aschieri D, Lazzeroni D, Rossi L, Biagi A, Binno SM, et al. Air pollution and out-of-hospital cardiac arrest risk: a 7-year study from a highly polluted area. Eur Heart J Acute Cardiovasc Care. 2023 Jun;12(12):810–7.

4. Zhao B, Johnston FH, Salimi F, Kurabayashi M, Negishi K. Short-term exposure to ambient fine particulate matter and out-of-hospital cardiac arrest: a nationwide case-crossover study in Japan. Lancet Planet Health. 2020 Jun;4(1):e15–23.

5. Dennekamp M, Akram M, Abramson MJ, Tonkin A, Sim MR, Fridman M, et al. Outdoor Air Pollution as a Trigger for Out-of-hospital Cardiac Arrests. Epidemiology. 2010 Jun;21(4):494–500.

6. Dai X, He X, Zhou Z, Chen J, Wei S, Chen R, et al. Short-term effects of air pollution on out-of-hospital cardiac arrest in Shenzhen, China. Int J Cardiol. 2015 Jun;192:56–60.

7. Yorifuji T, Suzuki E, Kashima S. Outdoor Air Pollution and Out-of-Hospital Cardiac Arrest in Okayama, Japan. J Occup Environ Med. 2014 Jun;56(10):1019–23.

8. Raza A, Bellander T, Bero-Bedada G, Dahlquist M, Hollenberg J, Jonsson M, et al. Short-term effects of air pollution on out-of-hospital cardiac arrest in Stockholm. Eur Heart J. 2014 Jun;35(13):861–8.

9. Dahlquist M, Frykman V, Hollenberg J, Jonsson M, Stafoggia M, Wellenius GA, et al. Short‐Term Ambient Air Pollution Exposure and Risk of Out‐of‐Hospital Cardiac Arrest in Sweden: A Nationwide Case‐Crossover Study. J Am Heart Assoc. 2023 Jun;12(21).

10. Kim JH, Hong J, Jung J, Im JS. Effect of meteorological factors and air pollutants on out-of-hospital cardiac arrests: a time series analysis. Heart. 2020 Jun;106(16):1218–27.

11. Ensor KB, Raun LH, Persse D. A Case-Crossover Analysis of Out-of-Hospital Cardiac Arrest and Air Pollution. Circulation. 2013 Jun;127(11):1192–9.

12. Silverman RA, Ito K, Freese J, Kaufman BJ, Claro D De, Braun J, et al. Association of Ambient Fine Particles With Out-of-Hospital Cardiac Arrests in New York City. Am J Epidemiol. 2010 Jun;172(8):917–23.

13. Cheng FJ, Wu KH, Hung SC, Lee KH, Lee CW, Liu KY, et al. Association between ambient air pollution and out-of-hospital cardiac arrest: are there potentially susceptible groups? J Expo Sci Environ Epidemiol. 2020 Jun;30(4):641–9.

14. Wichmann J, Folke F, Torp-Pedersen C, Lippert F, Ketzel M, Ellermann T, et al. Out-of-Hospital Cardiac Arrests and Outdoor Air Pollution Exposure in Copenhagen, Denmark. PLoS One. 2013 Jun;8(1):e53684.

15. Rosenthal FS, Kuisma M, Lanki T, Hussein T, Boyd J, Halonen JI, et al. Association of ozone and particulate air pollution with out-of-hospital cardiac arrest in Helsinki, Finland: Evidence for two different etiologies. J Expo Sci Environ Epidemiol. 2013 Jun;23(3):281–8.

16. Straney L, Finn J, Dennekamp M, Bremner A, Tonkin A, Jacobs I. Evaluating the impact of air pollution on the incidence of out-of-hospital cardiac arrest in the Perth Metropolitan Region: 2000–2010. J Epidemiol Community Health (1978). 2014 Jun;68(1):6–12.

17. Wang YC, Sung FC, Chen YJ, Cheng CP, Lin YK. Effects of extreme temperatures, fine particles and ozone on hourly ambulance dispatches. Science of The Total Environment. 2021 Jun;765:142706.

18. Kang SH, Heo J, Oh IY, Kim J, Lim WH, Cho Y, et al. Ambient air pollution and out-of-hospital cardiac arrest. Int J Cardiol. 2016 Jun;203:1086–92.

19. Zhao R, Chen S, Wang W, Huang J, Wang K, Liu L, et al. The impact of short-term exposure to air pollutants on the onset of out-of-hospital cardiac arrest: A systematic review and meta-analysis. Vol. 226, International Journal of Cardiology. 2017.

20. Sangkharat K, Fisher P, Thomas GN, Thornes J, Pope FD. The impact of air pollutants on ambulance dispatches: A systematic review and meta-analysis of acute effects. Vol. 254, Environmental Pollution. 2019.
